# LCRA predicts event-aligned canopy responses across unseen Qinling landscapes

**DOI:** 10.3389/fpls.2026.1955922

**Published:** 2026-10-28

**Authors:** Yuanyuan Shen, Xuyue Shen, Guozhen Liu, Tao Zhou, Chunyang Gu, Yan Wang, Donghui Zhang

**Affiliations:** 1 College of Biological and Environmental Engineering, Xi’an University, Xi’an, China; 2 Institute of Remote Sensing Satellite, China Academy of Space Technology, Beijing, China

**Keywords:** canopy greenness, compound hot-dry events, deep time-series prediction, enhanced vegetation index, matched landforms, phenology, Qinling Mountains, spatial cross-validation

## Abstract

**Introduction:**

Predicting event-aligned canopy variation in mountains requires integrating vegetation state, phenology, irregular satellite observations, and landform context. We asked whether a task-structured predictor could improve spatial transfer of short-horizon EVI responses across unseen Qinling landscapes.

**Methods:**

We studied 80 response-blind matched sets in the Qinling Mountains, each comprising one mapped landform and two same-event non-landslide controls, yielding 991 set-events and 2,973 member responses across 32 spatial blocks. The landform-conditioned canopy response aggregator (LCRA) integrated dated MODIS EVI with climate-season, terrain, canopy-state summaries, and matched-member relations to predict subsequent-minus-pre-event (S-P) and subsequent-minus-impact (S-I) EVI. Models were evaluated with block-exclusive outer folds, and nominal composite-start-window results were compared with a complete 16-day support analysis.

**Results:**

In the nominal analysis, the compact LCRA core ensemble achieved member *R*^2^ = 0.8219 versus 0.7964 for the EVI-state-plus-season linear reference Δ*R*^2^ = +0.0254; 95%; block × shared-onset interval 0.0037−0.0532; matched-contrast *R*^2^ was comparable (0.3684 versus 0.3677). Requiring complete 16-day input support to end before the target window yielded member *R*^2^ = 0.7810 in 80 sets and 0.7988 in all 90 eligible sets; in the latter, Δ*R*^2^ versus linear was +0.0240 −0.0037 to 0.0547), while MAE decreased by 0.0041 EVI (0.0002–0.0087). Validation-based variance scaling reduced primary-task 95% interval width by 38.6% and brought empirical coverage from 99.4% to 92.9%, with improved probabilistic scores. Observed responses showed seasonal sign reversal: mean S-P was +0.0710 EVI in May–June and −0.1814 and −0.1849 in July–August and September. Pre-event canopy state provided a second ordered axis; calendar-standardized severity responses were negative but non-monotonic. The regional landform contrast centered near zero, with bidirectional conditional spatial variation.

**Discussion:**

LCRA combines accurate spatial transfer with a phenology-centered account of event-aligned canopy trajectories. Complete temporal separation retained strong absolute accuracy, although relative *R*^2^ gains remained uncertain. These observational contrasts describe canopy variation rather than causal effects of hot–dry exposure.

## 1 Introduction

Mountain canopies do not follow a single short-term trajectory after compound hot–dry events. Their next observed greenness state is embedded in phenological phase, recent canopy condition, hydroclimatic exposure, and terrain, all of which vary sharply across the Qinling Mountains. Compound events combine thermal and moisture stress in ways that need not be represented by either driver alone (Zscheischler et al., 2020), while satellite evidence shows strong variation in canopy trajectories across biomes, hydroclimatic regimes, and event intensity (Yao et al., 2022; Liu et al., 2023; Li et al., 2024; Yang et al., 2024). Global assessments similarly connect vegetation stability to water availability and to the response definition used for evaluation (Smith et al., 2022; Ingrisch et al., 2023; Smith and Boers, 2023). The immediate scientific need is therefore predictive and spatial: identify the subsequent canopy-greenness window for locations not used to fit the model and resolve which vegetation and landscape contexts organize that movement. These studies together show that short-horizon EVI trajectories are jointly organized by phenological phase, antecedent canopy state, drought legacy, and hydroclimatic context (Gonçalves et al., 2020; Kato et al., 2021), with mountain terrain amplifying their spatial heterogeneity.

Mapped landslide terrain provides a contemporary ecological context characterized by substrate redistribution, altered drainage, slope exposure, and vegetation succession (Yunus et al., 2020; Rasigraf and Wagner, 2022). Because the inventory does not date every original mass movement, the polygons represent contemporary mapped landform units rather than dated disturbances. Each mapped landform was paired with two response-blind, same-event non-landslide control geometries sharing the 2020 dominant IGBP class and similar environmental settings. All three members shared the identified hot–dry event; the contrast quantifies differences associated with mapped-landform status among locations sharing the same event. The task jointly predicts all three member responses and the event-specific exposed-minus-control departure from the two-control reference.

The resulting event-centered remote-sensing task differs from a conventional dense time-series forecast. Alternating Terra and Aqua MODIS 16-day composites provide seven or eight dated observations over the 64-day input horizon, and their nominal epochs are irregular relative to event onset. Each example is a related triplet, and grouped evaluation preserves dependence among its three members; the dataset contains 991 set-events from 80 matched sets, 32 spatial blocks, and 281 shared onset dates. We define S−P as subsequent-window EVI relative to the pre-event state and pair it with S−I, the impact-to-subsequent transition that removes the shared pre-event term. These two window differences support complementary canopy-monitoring questions.

Recent sequence models supply demanding references for different aspects of this task. PatchTST learns channel-independent temporal patches (Nie et al., 2023), TimesNet reorganizes temporal variation in two dimensions (Wu et al., 2023), iTransformer treats variates as tokens (Liu et al., 2024); TimeMixer mixes multiscale components (Wang et al., 2024a), ModernTCN applies large-kernel temporal convolutions (Luo and Wang, 2024), and TimeXer separates endogenous and exogenous information (Wang et al., 2024b). WPMixer and xPatch add multi-resolution mixing and seasonal–trend decomposition (Murad et al., 2025; Stitsyuk and Choi, 2025). The present task instead requires joint representation of one mapped landform, two matched controls, and member/contrast outputs. Compact tabular predictors supply an additional reference based on canopy state, climate, and terrain without explicit dated order or matched-member geometry.

We designed the landform-conditioned canopy response aggregator (LCRA) around that missing task structure. A learned summary-and-context direct branch anchors the prediction in observed pre/impact canopy state, climate-season, and static context; a time-aware masked update retains dated EVI order; a matched-relational update exchanges information among the exposed member and two controls; and a probabilistic decoder produces member means and input-dependent variances. The training protocol combines member and matched-contrast objectives, while evaluation assigns complete spatial blocks exclusively to outer folds. Compact K = 1 and extended K = 4 configurations expose the accuracy–cost frontier of the same operator family, and validation-selected iTransformer fusion supplies a separate accuracy layer.

The algorithmic question is whether this structure improves member-level canopy prediction across held-out Qinling blocks relative to two conventional models and eight recent deep architectures, and which inputs, components, objective terms, and stage depth support that result. The vegetation questions then ask how season and pre-event canopy state organize S−P and S−I, how severity and terrain refine those dominant patterns after calendar adjustment, and where mapped landforms depart conditionally from their matched controls when the regional mean contrast is near zero.

The study makes three contributions. First, it establishes a phenology-aware matched-landform prediction task coupling S−P and S−I with complete-triplet, block-exclusive spatial evaluation. Second, it introduces LCRA as a task-structured predictor that integrates irregular dated EVI, climate–season, terrain, and matched-member structure to jointly predict member responses, matched contrasts, and input-dependent uncertainty; broad benchmarking, ablation, loss transfer, seed replication, and stage-depth analysis localize its gains. Third, it links spatially transferred predictions to a quantitative vegetation account of seasonal sign reversal, canopy-state gradients, calendar-standardized severity, terrain organization, and bidirectional conditional landform departures across the Qinling Mountains.

## 2 Materials and methods

### 2.1 Study system, matched design, and canopy-response tasks

#### 2.1.1 Study region and matched-landform design

The study domain spans the Qinling Mountains and adjacent systems between approximately 102.4°–113.7° E and 30.7°–35.5°N (**Figures 1A, B**). The region bridges warm-temperate and northern-subtropical environments and contains strong gradients in elevation, slope, aspect, vegetation, and hydroclimatic exposure. Mapped landslide polygons were obtained from the open Qinling inventory (Feng et al., 2024). The polygons supplied present-day landform geometries, and the canopy-response task was estimated independently of susceptibility labels, external model predictions, and inferred disturbance ages. A predefined Qinling study-domain polygon delimited mapped-landform and matched-control candidates before response construction; its outline represents the analytical mountain domain rather than an administrative boundary or a feature inferred from canopy outcomes.

**Figure 1:**
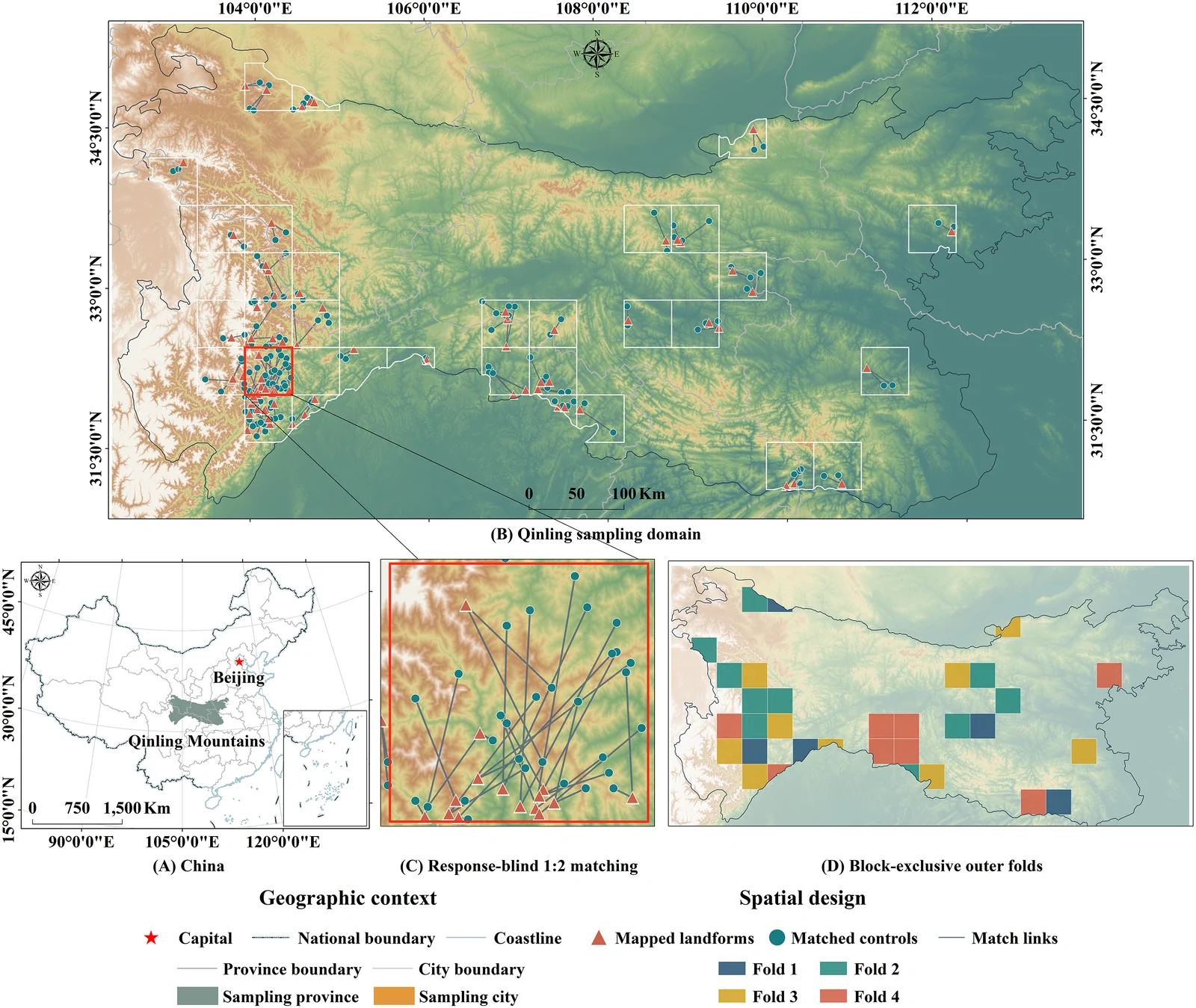
Geographic context and spatial design of the matched Qinling canopy-response cohort. **(A)** Qinling location in China. **(B)** Copernicus DEM GLO-30 elevation across the predefined Qinling study domain (displayed range 24–4,942 m), with the 80 mapped landforms, 160 same-event matched non-landslide controls, match links, and occupied 50-km blocks; the red box marks **(C)**. The irregular outline delimits the response-blind candidate domain and is not an administrative boundary. **(C)** Response-blind 1:2 matching. **(D)** Four block-exclusive outer folds; matched sets remain intact within folds. **(B–D)** use the common Albers equal-area projection.

Each mapped landform was paired with two same-event matched non-landslide control geometries through a response-blind global assignment (**Figure 1C**). Candidate pairs shared the same 50-km block and primary IGBP class. Static land-cover matching used the 2020 MCD12Q1 Collection 6.1 LC_Type1 and QC layers (500 m; DOI: 10.5067/MODIS/MCD12Q1.061), retaining QC = 0 pixels and plant-domain IGBP classes 1–10. The 2020 layer was prespecified as one response-blind landscape anchor for all event years, avoiding year-specific rematching and preserving a common geometric reference. Land-cover similarity therefore refers specifically to the dominant IGBP class in the 2020 matching layer, not to demonstrated agreement in each event year. Temporal stability of this class across individual event years was not evaluated. Event-aligned MODIS EVI and ERA5-Land supplied time-varying canopy and climate information. Twelve continuous matching variables were natural-log polygon area, perimeter divided by square-root area, primary-class IGBP area fraction, mean elevation, slope, northness, eastness, May–September mean daily-maximum temperature, warm-season soil-water layers 2 and 3, and log-one-plus distance to the nearest road and HydroRIVERS segment. Terrain covariates were derived from a Copernicus DEM GLO-30 China mosaic reprojected to the 30-m WGS84 Albers grid; horn 3 × 3 slope and aspect were then calculated. Road proximity used the Geofabrik China OpenStreetMap extract dated July 20, 2026, and river proximity used HydroRIVERS v1.0 Asia linework. Each variable was centered by the median over the eligible exposed universe and scaled by 1.4826 × MAD, with IQR/1.349 and sample SD as zero-scale fallbacks. Pairwise edges required an absolute robust-z difference no larger than 0.75 for the 11 geometry, terrain, climate, and distance features and 1.5 for primary-class area fraction. The 0.75 caliper was the smallest response-blind feasibility expansion from an initial 0.50 setting that yielded a complete assignment; 1.50 was the prespecified feature-specific caliper for primary IGBP-class fraction. Both values were set during match construction before response extraction. Robust Mahalanobis distance based on a minimum-covariance-determinant estimate ranked candidate edges.

The matching procedure first identified 160 exact two-control sets under non-reuse, non-overlap, and aggregate first- and second-moment balance constraints. A second response-blind optimization retained 110 sets under the same balance criteria; source-pair Mahalanobis cost served as the final tie-breaker. Controls were prohibited from overlapping one another or the accessible inventory and its 300-m exclusion buffer. This pool was defined before canopy-response extraction. Direct MODIS EVI availability subsequently yielded 90 sets with finite targets, from which the 80-set cohort was formed as described below.

Blocks were defined in an Albers equal-area CRS (+proj=aea +lat_1 = 25 +lat_2 = 47 +lat_0 = 0 +lon_0 = 105 +datum=WGS84) with 50,000-m cells and origin x0 = −250,000 m, y0 = 3,250,000 m; centroid floor indices generated the block IDs. The response-blind block-to-fold map was constructed once over the eligible exposed universe: blocks were ordered by descending unit count, with a fixed salted SHA-256 value and block ID as deterministic tie-breakers and then assigned to the fold with the lowest current unit load, block load, and fold index. The analyzed cohort retained the resulting fold 1–4 assignments (**Figure 1D**).

The primary 80-set benchmark used a prespecified allocation from the 90 sets with finite targets to hold fold-wise training size constant across model, ablation, and loss-transfer comparisons. Within each preassigned outer fold, unit-ID ordering and quotas of 22, 20, 22, and 16 selected sets before inspection of canopy responses. The complete 90-set sensitivity evaluated member-level prediction across all finite-target-eligible sets under the complete-support rule. The primary cohort contains 80 mapped landforms, 160 controls, and 32 occupied 50-km blocks (**Figures 1B,D**). Across all 12 prespecified matching variables, 10 had |SMD|<0.10 and all had |SMD|<0.20. The largest values were 0.148 for primary IGBP-class fraction and 0.108 for mean slope; the remaining 10 were ≤0.055 (**Supplementary Table S6**). Median exposed–control centroid distance was 15.8 km. The complete-row swap test establishes row-permutation equivariance for the explicitly encoded three-member input. **Table 1** summarizes cohort flow, spatial design, event support, observation windows, and dual response tasks.

**Table 1:** Study design, event windows, phenology encoding, dual targets, and analytical support.

Component	Study specification	Observed support
Matched cohort	Response-blind 1:2 matching; 90 finite-target-eligible sets; primary 80-set cohort fixed by within-fold unit-ID ordering and quotas 22/20/22/16	80 sets; 240 geometries; all 12 matching covariates in **Supplementary Table S6** (10 |SMD|<0.10; all<0.20; maximum 0.148)
Spatial grid/evaluation	50-km Albers equal-area blocks; four block-exclusive outer folds; complete sets remain intact	32 occupied blocks; origin (−250 km, 3,250 km)
Event thresholds	Local-calendar q80 daily-maximum temperature and q20 thickness-weighted soil moisture; 2001–2019 reference; centered ±7-day samples	ERA5-Land; May–September
Matched event support	≥2 consecutive days; each of 3 members ≥50% area exposed; ≤32-day run; 40-day declustering; ≥10 events across ≥8 years per set	281 onsets; 991 set-events
Remote sensing	Direct MOD13Q1.061/MYD13Q1.061 EVI at 250 m; product composite-start epochs with 16-day support; product-valid weights and valid-cell fractions retained; HLS/Fmask used only to define the response-blind eligibility calendar	2013–2020 and 2022–2025; 2021 excluded by the prespecified pre-response eligibility rule
Input horizon	Primary analysis: 32-day pre-event plus 32-day impact interval; conservative sensitivity retains only complete 16-day composite support ending before target day 32	Primary analysis: 2,662 member-events with 8 observations and 311 with 7; complete-support 80-set analysis retained 17,974/23,473 input composite records; complete-support 90-set analysis retained 20,266/26,464; maximum support-end day 31 in both
Primary task	Subsequent minus pre-event mean EVI (S−P)	2,973 member-event targets
Sensitivity task	Subsequent minus impact mean EVI (S−I); no shared pre-event reference	2,973 member-event targets
Phenology	Onset day-of-year sine and cosine, known at event onset	Five onset months; 12 represented years

The primary analysis uses the response-blind 80-set cohort; complete-support results for the 80- and 90-set cohorts are reported in **Supplementary Table S7**.

#### 2.1.2 Compound hot–dry events and event-centered EVI

Compound events were derived from ERA5-Land temperature and soil-moisture records, which provide an internally consistent hourly description of land-surface water and energy conditions (Muñoz-Sabater, 2019; Munoz-Sabater et al., 2021). Local-calendar thresholds were estimated over 2001–2019 from centered ±7-day samples: local daily maximum 2-m temperature had to be strictly above its 0.80 quantile, and the 0.21/0.72-m thickness-weighted mean of soil–water layers 2–3 had to be strictly below its 0.20 quantile. Daily compound masks used eight-neighbor components with at least four cells, maximum cross-day gap of 2 days, and cell-set Jaccard overlap of at least 0.25.

A matched event required at least two consecutive compound days, with at least 50% area-weighted exposure in the mapped landform and both controls. Simultaneous runs were capped at 32 days; onsets separated by fewer than 40 days were declustered by retaining the earlier onset. Sets contributed when they contained at least 10 events spanning at least 8 years. Calendar eligibility required a complete, response-blind Harmonized Landsat Sentinel-2 (HLS) Fmask calendar before any MODIS response was derived. HLS/Fmask served only to define eligible climate-event dates; neither HLS reflectance nor Fmask entered the MODIS EVI predictor tensors or targets. The prespecified HLS/Fmask calendar could not be completed for 2021, so that year was excluded before MODIS response extraction; the analytical period comprises May–September 2013–2020 and 2022–2025. The resulting descriptors were simultaneous run length, maximum and accumulated temperature exceedance, integrated soil-moisture deficit, and mean and minimum affected-area fraction. EVI did not define events.

Canopy greenness was represented by direct MOD13Q1.061 and MYD13Q1.061 EVI at 250-m resolution (Didan, 2021b, a). EVI retains sensitivity in dense vegetation while reducing several atmospheric and canopy-background effects relative to a simple ratio index (Huete et al., 2002). Product values were scaled by 0.0001 and aggregated over each member geometry with fixed fractional vegetation-area weights. Response extraction used the direct EVI layer and retained product-valid weights and valid-cell fractions; no separate reliability threshold was applied. Terra/Aqua identity, the product’s nominal Ayyyyddd composite epoch, valid weight, and valid-cell fraction were retained. Each response window required at least two composites. The model input contained seven composites for 311 member-events and eight for 2,662 member-events; missing input slots remained unfilled. Fractional-area weights determine each intersecting pixel’s contribution to polygon-level EVI but do not spectrally unmix a 250-m cell. Physical support was therefore described by the number of intersecting MODIS cells and by the Kish-effective cell count derived from the fractional weights; both measures were related to event-level absolute matched contrast (|Δ|) using Spearman association and support-quartile summaries. Retained valid-cell fractions were high: the minimum was 0.8261 among 991 mapped-landform member-events and 0.7037 among 1,982 control member-events; no mapped-landform observation and one control observation fell below the descriptive 0.80 reference. All observations were retained (**Supplementary Table S8**).

Equation 1 partitions each event at its onset into non-overlapping 32-day pre-event, impact, and subsequent half-open intervals measured in nominal composite-epoch days; *e* indexes the event and *t*_0_ denotes its onset.

(1) $$P_{e}=\left[t_{0,e}-32,t_{0,e}\right),\;{ℐ}_{e}=\left[t_{0,e},t_{0,e}+32\right),\;S_{e}=\left[t_{0,e}+32,t_{0,e}+64\right)$$

Equation 2 computes the fixed-weight EVI mean for member *m*, event *e*, and window *W*; *n* is the number of valid composites, *E* is scaled EVI, and omega is the retained fractional vegetation-area weight. The denominator is positive because each response window requires at least two composites.

(2) $${\bar{E}}_{m,e}^{W}=\frac{\sum_{j=1}^{n_{m,e}^{W}}\omega_{m,e,j}^{W}E_{m,e,j}^{W}}{\sum_{j=1}^{n_{m,e}^{W}}\omega_{m,e,j}^{W}},\;W\in \left\{P,ℐ,S\right\}$$

The input and response windows in the primary analysis were assigned by MODIS product composite-start epoch. Input starts fell in the pre-event and impact intervals, and composite starts in the subsequent interval formed the response. Across the 2,973 member-events, the final input start occurred between event days +24 and +31. Because each MODIS value summarizes 16 days beginning at its product start date, nominal-window assignment allows the final input support to extend 8–15 days beyond day +31. A support-window-conservative no-overlap sensitivity (hereafter complete-support sensitivity) therefore retained only input composites starting no later than day +16, so every retained 16-day support ended by day +31 before the target began at day +32. In the 80-set cohort, this rule retained 17,974 of 23,473 input composite records, preserved all 991 events, and produced zero boundary violations. This sensitivity evaluates complete product-level composite support rather than selected within-composite observation dates. The nominal-window results characterize the composite-start-defined monitoring task; claims about temporally separated prediction are based on the complete-support analysis. **Figure 2** summarizes both temporal specifications and the S−P/S−I responses.

**Figure 2:**
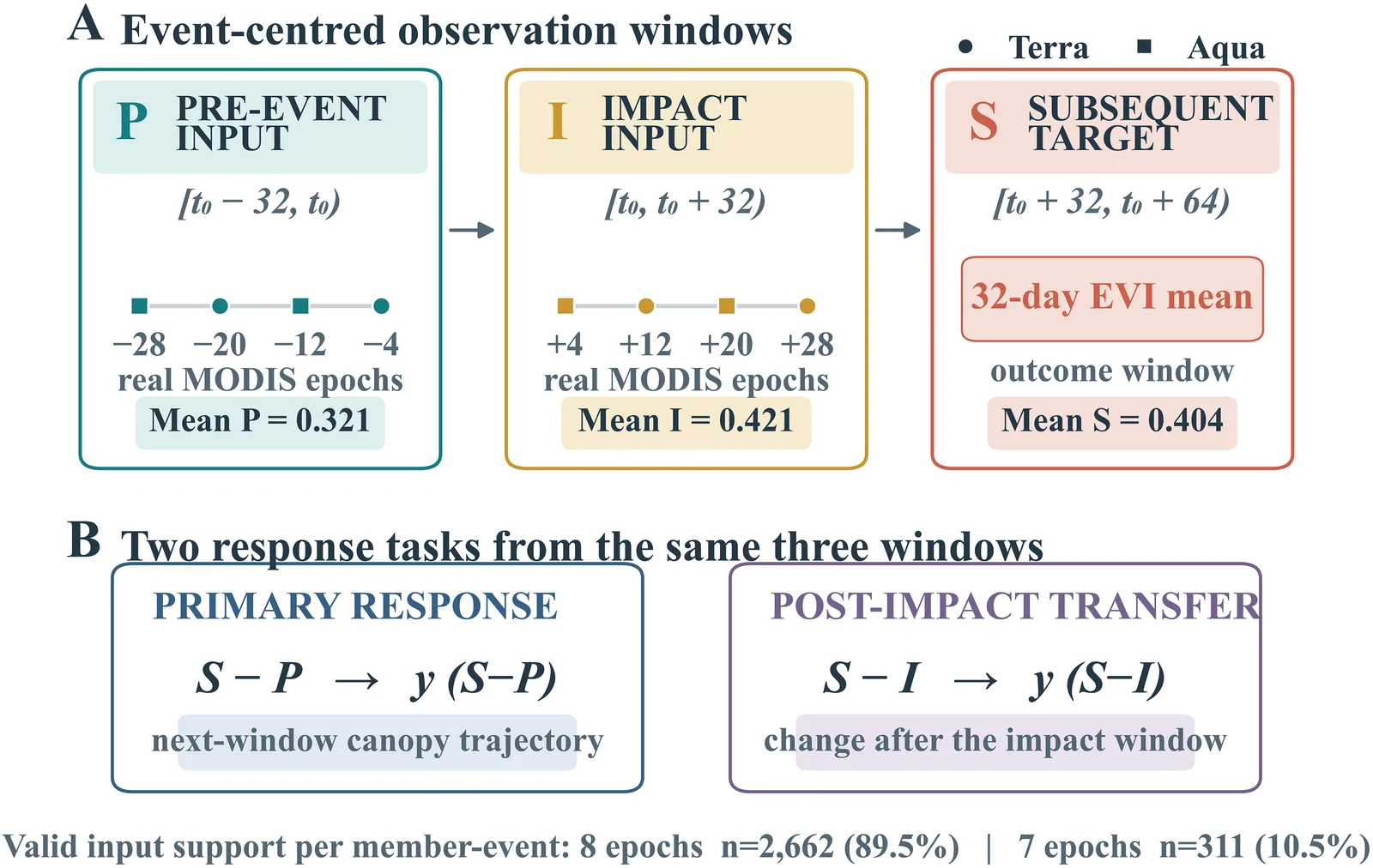
Event-aligned MODIS sampling and response construction. **(A)** Pre-event (P), impact (I), and subsequent (S) 32-day windows, with Terra and Aqua product composite starts and the subsequent EVI aggregate. The complete-support sensitivity retains input composites starting no later than day +16, so their complete 16-day support ends by day +31 before the target begins at day +32; it is defined by product-level support rather than selected within-composite observation dates. **(B)** The two responses constructed from the same windows: primary S−P and post-impact S−I. The support line reports the observed seven- and eight-composite member-event counts.

#### 2.1.3 Dual response tasks, seasonal phase, and matched estimands

Equation 3 jointly defines the primary S−P response, the observed I−P impact anomaly, and the alternate S−I response for member *m* and event *e* and states the exact identity that removes their shared pre-event reference.

(3) $$\begin{array}{c} y_{m,e}^{SP}={\bar{\text{ E}}}_{m,e}^{S}-{\bar{\text{ E}}}_{m,e}^{P}\\ a_{m,e}^{IP}={\bar{\text{ E}}}_{m,e}^{ℐ}-{\bar{\text{ E}}}_{m,e}^{P}\\ y_{m,e}^{SI}={\bar{\text{ E}}}_{m,e}^{S}-{\bar{\text{ E}}}_{m,e}^{ℐ}\\ y_{m,e}^{SI}=y_{m,e}^{SP}-a_{m,e}^{IP} \end{array}$$

Thus, S−I algebraically removes the shared pre-event reference that links S−P and I−P. S−P is a pre-state-relative monitoring endpoint, and S−I is an impact-to-subsequent transition endpoint; together they quantify complementary observed canopy transitions across the three fixed windows. Positive responses indicate a subsequent mean greener than the comparison window, and negative responses indicate a lower subsequent mean.

Equation 4 defines the event-specific matched contrast; *q* indexes the S−P or S−I task, member 0 is the mapped landform, members 1 and 2 are its controls, and the two control responses receive equal weight.

(4) $$\Delta_{e}^{q}=y_{0,e}^{q}-\frac{y_{1,e}^{q}+y_{2,e}^{q}}{2},\;q\in \left\{SP,SI\right\}$$

Member 0 is the mapped landform and members 1–2 are its same-event matched non-landslide controls. The regional contrast summarizes the distribution center; contrast prediction tests whether event-specific departures around that center are learnable. No non-event temporal counterfactual was included. Both the member responses and matched contrasts therefore describe event-aligned canopy transitions rather than causal effects of hot–dry exposure.

Equation 5 maps onset day of year to sine and cosine coordinates on the 365.25-day seasonal cycle; *d* denotes onset day for event *e*, and the paired coordinates avoid a year-end discontinuity.

(5) $$s_{e}=sin\left(\frac{2\pi \left(d_{e}-1\right)}{365.25}\right),\;c_{e}=cos\left(\frac{2\pi \left(d_{e}-1\right)}{365.25}\right)$$

The two values were repeated across the matched members and combined with six event-climate descriptors. S−P and S−I were trained and evaluated as separate tasks. Fold-specific counts of blocks, matched sets, set-events, member-events, and unique onsets are reported in **Supplementary Table S4**.

### 2.2 LCRA representation and matched-relational learning

#### 2.2.1 Inputs, normalization, and deterministic summaries

Equation 6 defines the eight-dimensional observation token for member m, event e, and nominal composite j.

(6) $$x_{m,e,j}={\left[E_{m,e,j},q_{m,e,j},u_{m,e,j},\ell_{m,e,j},\tau_{m,e,j},r_{m,e,j},p_{m,e,j},i_{m,e,j}\right]}^{⊤}$$

In Equation 6, the eight entries denote EVI, valid weight, valid-cell fraction, sensor identity, nominal-epoch relative day, inter-epoch gap, pre-window flag, and impact-window flag, respectively. Stacking these tokens produces a 3 × 8 × 8 temporal tensor; the climate-season and static tensors each have shape 3 × 8. The leading dimension indexes the mapped landform and its two controls.

The static descriptors were native geometry-support count, fractional-area sum, mean elevation, mean slope, northness, eastness, role indicator, and within-triplet position. Inputs followed each comparator’s declared design: the linear reference used compact EVI-state and season terms, extra trees used flattened temporal/context features and deterministic summaries, the eight deep adapters used the 3 × 8 × 8 temporal tensor with their documented climate/static task heads, and LCRA used X, C, G and the summaries in Equation 10. These static geometry fields are distinct from the intersecting MODIS 250-m physical-cell support summarized in **Supplementary Table S8**.

Equation 7 standardizes each continuous input or target *z* with mean and standard deviation estimated only from the inner-fitting blocks of outer fold *F*; the displayed subscripts identify the variable and fold, and the 10^−5^ floor prevents division by zero.

(7) $$\tilde{z}=\frac{z-\mu_{z,F}}{\max\left(\sigma_{z,F},10^{-5}\right)}$$

Equation 8 defines the masked mean of a scalar sequence *v*, where the binary mask *b* equals one for a valid observation; the denominator clamp gives a zero result for an all-masked sequence.

(8) $$ℳ\left(v,b\right)=\frac{\sum_{j}b_{j}v_{j}}{\max\left(\sum_{j}b_{j},1\right)}$$

Equation 9 defines the masked least-squares EVI slope for member *m* and event *e*; the overbarred relative-day and EVI terms are the masked means from Equation 8, and 10^−4^ is a numerical denominator floor.

(9) $$\beta_{m,e}=\frac{\sum_{j}b_{j}\left(\tau_{j}-\bar{\tau }\right)\left(E_{j}-\bar{E}\right)}{\max\left(\sum_{j}b_{j}{\left(\tau_{j}-\bar{\tau }\right)}^{2},10^{-4}\right)}$$

Equation 10 first assembles the 12-dimensional deterministic summary *q* and then concatenates it with the eight-dimensional climate-season vector *C* and eight-dimensional static vector *G*. The resulting initializer input *f* has 28 channels for every member-event. **Supplementary Table S5** shows a list of the complete input-variable dictionary and its placement within the temporal, climate–season, and static tensors.

(10) $$\begin{array}{c} q_{m,e}={\left[{\bar{E}}^{P},{\bar{E}}^{ℐ},{\bar{E}}^{ℐ}-{\bar{E}}^{P},E_{\min}^{P},E_{\min}^{ℐ},E_{\max}^{P},E_{\max}^{ℐ},E_{\min}^{ℐ}-{\bar{E}}^{P},\bar{E},\beta ,n_{P}/8,n_{ℐ}/8\right]}^{⊤}\\ f_{m,e}={\left[q_{m,e}^{⊤},C_{m,e}^{⊤},G_{m,e}^{⊤}\right]}^{⊤} \end{array}$$

#### 2.2.2 LCRA initializer and time-aware canopy memory

Equation 11 defines the learned direct response and 64-dimensional initial member state. The mean branch maps 28 to 128 to 64 to 1 with GELU and dropout 0.08, whereas the initializer maps 28 to 128 to 64 with GELU and layer normalization; the additive initial-state vector is learned and shared.

(11) $$\begin{array}{c} b_{m,e}=F_{mean}\left(f_{m,e}\right)\\ h_{m,e}^{\left(0\right)}=h_{0}+F_{init}\left(f_{m,e}\right) \end{array}$$

This combination lets the recurrent path learn a residual correction around a learned summary-and-context base prediction rather than rebuilding the entire response from a short sequence.

Equation 12 defines one masked recurrent scan: the input layer projects each eight-channel standardized token to 64 channels, the GRUCell proposes a dated update, and the binary mask either accepts that proposal or carries the preceding state through an invalid slot.

(12) $$\begin{array}{c} z_{m,e,j}=\text{LN}\left(\text{GELU}\left(W_{x}{\tilde{x}}_{m,e,j}+b_{x}\right)\right)\\ {\hat{h}}_{j}=\text{GRUCell}\left(z_{j},h_{j-1}\right)\\ h_{j}=b_{j}{\hat{h}}_{j}+\left(1-b_{j}\right)h_{j-1} \end{array}$$

Equation 13 defines the 64-dimensional summary projection, temporal gate, and gated residual refiner. The summary MLP maps 12 to 64 to 64, the gate maps its 192-channel concatenated state, proposal, and summary to 64 channels, and dropout is 0.08.

(13) $$\begin{array}{c} r_{m,e}=W_{r2}\text{GELU}\left(W_{r1}q_{m,e}+b_{r1}\right)+b_{r2}\\ {ɡ}_{T}=\sigma \left(W_{T2}\text{GELU}\left(W_{T1}\left[h,\hat{h},r\right]+b_{T1}\right)+b_{T2}\right)\\ T\left(h\right)=\text{LN}\left(h+\text{Dropout}\left(g_{T}⊙\left(\hat{h}-h\right)\right)+r\right) \end{array}$$

Nominal-epoch relative days and inter-epoch gaps enter the token, making the refiner explicitly time-aware.

#### 2.2.3 Matched-relational update

Equation 14 maps the 16-channel climate-season/static concatenation to a 64-dimensional context and injects it into each member state; the context MLP maps 16 to 64 to 64.

(14) $$\begin{array}{c} k_{m,e}=W_{k2}\text{GELU}\left(W_{k1}\left[C_{m,e},G_{m,e}\right]+b_{k1}\right)+b_{k2}\\ u_{m,e}=\text{LN}\left(h_{m,e}+k_{m,e}\right) \end{array}$$

Equation 15 defines four-head self-attention over the three matched members: the query, key, and value tensors remain 64-dimensional, each head has width 16, the softmax operates across members, and the output projection restores the concatenated heads before residual layer normalization.

(15) $$\begin{array}{c} Q=uW_{Q},\;K=uW_{K},\;V=uW_{V}\\ A_{r}=\mathrm{softmax}\left(\frac{Q_{r}K_{r}^{⊤}}{\sqrt{d_{h}}}\right)V_{r}\\ \tilde{u}=\text{LN}\left(u+\mathrm{Concat}\left(A_{1},\ldots ,A_{H}\right)W_{O}\right) \end{array}$$

Equation 16 forms the symmetric two-control mean and broadcasts the exposed-minus-control 64-vector to all three members; tensor-product replication along the three-member axis makes this explicit term invariant to swapping the two complete control rows.

(16) $$\begin{array}{c} {\bar{u}}_{C}=\frac{{\tilde{u}}_{1}+{\tilde{u}}_{2}}{2}\\ d=1_{3}⊗\left({\tilde{u}}_{0}-{\bar{u}}_{C}\right) \end{array}$$

Equation 17 defines the 64-dimensional relational gate and residual update. The gate maps the 192-channel concatenation of current, attended, and contrast states to 64 channels, and the feed-forward path maps 64 to 192 to 64 with GELU and dropout 0.08.

(17) $$\begin{array}{c} g_{R}=\sigma \left(W_{R2}\text{GELU}\left(W_{R1}\left[u,\tilde{u},d\right]+b_{R1}\right)+b_{R2}\right)\\ R\left(u\right)=\text{LN}\left(u+g_{R}⊙\left(\tilde{u}-u\right)+F_{FF}\left(\tilde{u}\right)\right) \end{array}$$

Equation 16 makes the explicit two-control summary invariant to their order. A complete-row swap test exchanged the two control rows, including their position codes, and mapped the outputs back to the original order. The maximum and mean absolute prediction differences were 8.94 × 10^−8^ and 6.37 × 10^−9^, establishing complete-row swap equivariance of the predictor.

#### 2.2.4 Stage depth, response decoder, and probabilistic output

Equation 18 defines stage *k*: the shared temporal operator is applied twice, context is injected once, and the shared relational operator is applied twice before the stage state is returned. A 64-dimensional learned embedding provides the stage-specific term, and the evaluated depths are *K* = 1 and *K* = 4.

(18) $$t_{e}^{\left(k\right)}=T^{\left[2\right]}\left(h_{e}^{\left(k-1\right)}+\eta_{k}\right),\;u_{e}^{\left(k,0\right)}=\text{LN}\left(t_{e}^{\left(k\right)}+k_{e}\right),\;u_{e}^{\left(k,s\right)}=ℛ\left(u_{e}^{\left(k,s-1\right)}\right),\;s=1,2,\;h_{e}^{\left(k\right)}=u_{e}^{\left(k,2\right)}$$

Here T[2] = T∘T denotes two complete masked GRU/gated passes. Context *k*_e_ from Equation 14 is injected once before the relational loop, and *R* denotes one attention/contrast-gate/feed-forward update from Equations 15–17; the second update receives the first update’s state. The same temporal-refiner and relational-refiner weights are reused within their two passes and across evaluated stages; the learned embedding assigns a distinct 64-dimensional vector to each stage. The evaluated configuration used hidden size 64, four attention heads, two temporal passes, two relational passes, and dropout 0.08. Depths *K* = 1 and *K* = 4 were trained and evaluated independently. *K* = 1 defines the compact Pareto configuration supported by complete out-of-fold accuracy, *S*−*I* point estimates, and computational cost; *K* = 4 provides the highest primary accuracy. Inner validation selected checkpoints separately for each fixed *K*, and the depth comparison uses their complete out-of-fold results.

Equation 19 maps the 64-dimensional stage state through layer normalization and a 64-to-64-to-32 GELU decoder to the shared representation *v* used by both output heads.

(19) $$v_{m,e}^{\left(k\right)}=\text{GELU}\left(W_{v2}\text{GELU}\left(W_{v1}\text{LN}\left(h_{m,e}^{\left(k\right)}\right)+b_{v1}\right)+b_{v2}\right)$$

Equation 20 adds the learned 32-channel residual mean to the direct branch and produces a clamped log variance. Clipping the log variance to [−8, 4] defines the displayed standard deviation on the standardized target scale, after which the stored fitting-partition scale restores EVI units. The complete flow from matched-member inputs to joint response, contrast, and probabilistic outputs is summarized in **Figure 3**.

**Figure 3:**
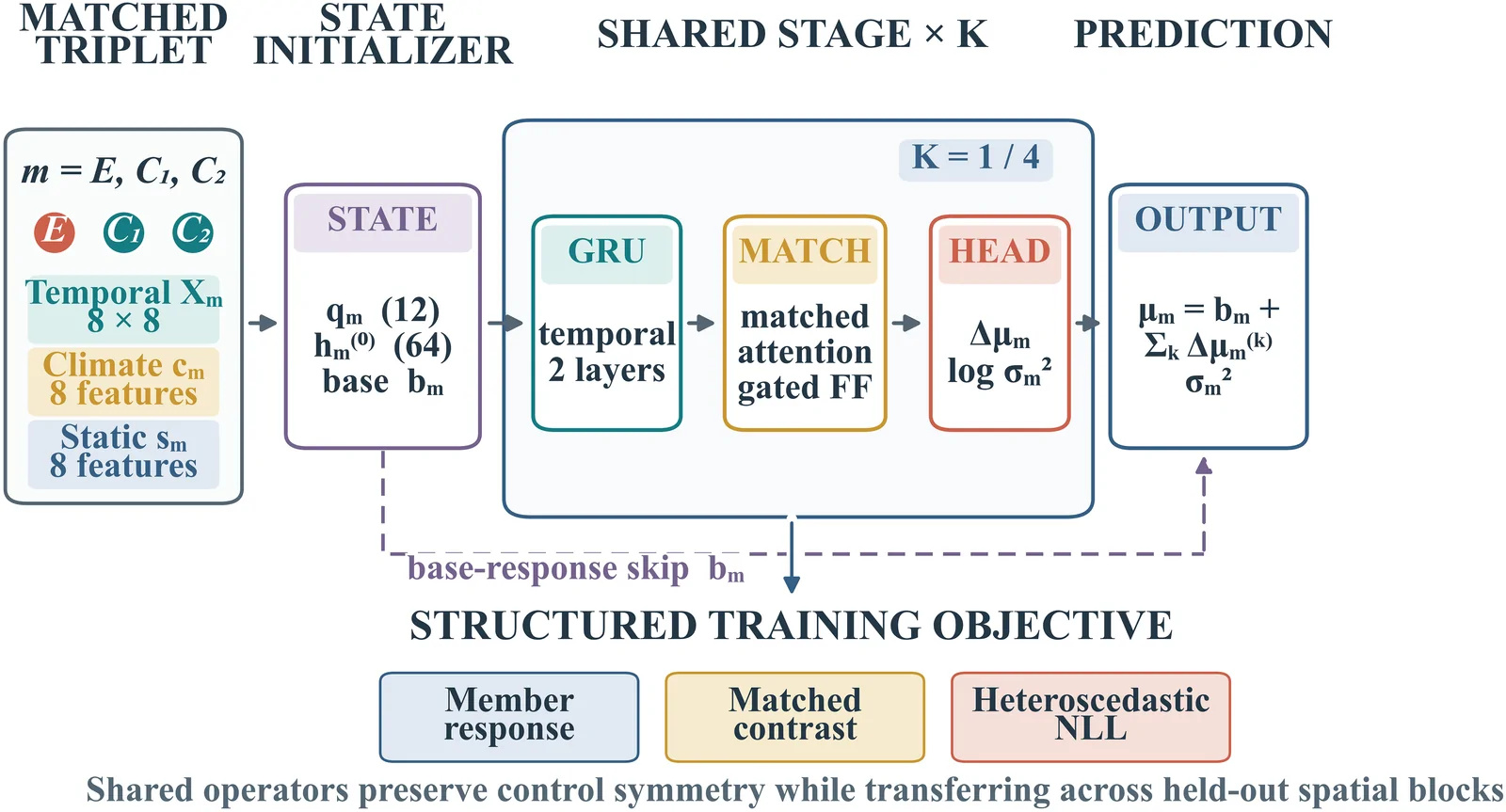
LCRA information flow. Solid arrows trace the memberwise path from the matched set through state initialization, recurrent memory, matched attention, decoding, and prediction. The dashed arrow carries the direct base response to the prediction, and the downward arrow links the shared stage to the joint member-response, matched-contrast, and heteroscedastic-NLL objective. K = 1 and K = 4 are independently trained configurations.

(20) $$\begin{array}{c} {\hat{y}}_{m,e}^{\left(k\right)}=b_{m,e}+w_{\mu }^{⊤}v_{m,e}^{\left(k\right)}+b_{\mu }\\ \ell_{m,e}^{\left(k\right)}=\text{clip}\left(w_{\ell }^{⊤}v_{m,e}^{\left(k\right)}+b_{\ell },-8,4\right),\;{\hat{\sigma }}_{m,e}^{\left(k\right)}=exp\left(\ell_{m,e}^{\left(k\right)}/2\right) \end{array}$$

### 2.3 Comparators, training, and spatial evaluation

#### 2.3.1 Training objective, fusion, and seed ensemble

Equation 21 defines the final-stage member-response mean-squared error, averaged over *B* complete matched events and their three members.

(21) $${ℒ}_{resp}=\frac{1}{3B}\sum_{e=1}^{B}\sum_{m=0}^{2}{\left({\hat{y}}_{m,e}^{\left(K\right)}-y_{m,e}\right)}^{2}$$

Equation 22 defines the predicted matched contrast and its event-averaged mean-squared error, using the same exposed-minus-two-control estimand as Equation 4.

(22) $${\hat{\Delta }}_{e}^{\left(K\right)}={\hat{y}}_{0,e}^{\left(K\right)}-\frac{{\hat{y}}_{1,e}^{\left(K\right)}+{\hat{y}}_{2,e}^{\left(K\right)}}{2},\;{ℒ}_{con}=\frac{1}{B}\sum_{e=1}^{B}{\left({\hat{\Delta }}_{e}^{\left(K\right)}-\Delta_{e}\right)}^{2}$$

Equation 23 defines the heteroscedastic Gaussian negative log-likelihood on the standardized target scale, where the displayed ell term is the predicted log variance; the additive log(2π)/2 constant is omitted because it does not affect optimization.

(23) $${ℒ}_{NLL}=\frac{1}{6B}\sum_{e=1}^{B}\sum_{m=0}^{2}\left[\ell_{m,e}^{\left(K\right)}+{\left(y_{m,e}-{\hat{y}}_{m,e}^{\left(K\right)}\right)}^{2}exp\left(-\ell_{m,e}^{\left(K\right)}\right)\right]$$

Equation 24 combines final response, stage-wise auxiliary response, matched-contrast, and negative-log-likelihood terms. The stage-specific response term reuses Equation 21, the stage weights are normalized, and the three lambda coefficients control the auxiliary contributions.

(24) $$ℒ={ℒ}_{resp}^{\left(K\right)}+\lambda_{D}\sum_{k=1}^{K}\alpha_{k}{ℒ}_{resp}^{\left(k\right)}+\lambda_{C}{ℒ}_{con}+\lambda_{N}{ℒ}_{NLL}$$

Here *α*_k_ increases linearly from 0.4 to 1.0 and is normalized across stages; the stage-wise auxiliary sum includes the final stage. The four-stage complete objective used λ*_D_* = 0.20, λ*_C_* = 0.20, and λ*_N_* = 0.02. For *K* = 1, stage-wise supervision is redundant and λ*_D_* was set to zero; λ*_C_* and λ*_N_* remained 0.20 and 0.02, respectively. These coefficients were held constant across the reported experiments. Target means were inverse-transformed with the fitting-partition target mean and standard deviation; target-scale variances were obtained by multiplying standardized variances by the squared fitting-partition target standard deviation. Models were optimized with AdamW, learning rate 8 × 10^−4^, weight decay 2 × 10^−4^, batch size 64, maximum 250 epochs, cosine annealing to 5% of the initial learning rate, gradient-norm clipping at 3.0, and early-stopping patience 45 with a 10^−5^ minimum validation improvement.

Equation 25 defines a member-wise convex fusion between the LCRA prediction and iTransformer prediction, with a shared weight between zero and one within each outer fold.

(25) $${\hat{y}}_{e}^{F}\left(w\right)=\left(1-w\right){\hat{y}}_{e}^{L}+w{\hat{y}}_{e}^{I},\;0\leq w\leq 1$$

Equation 26 selects the fold-specific fusion weight by minimizing RMSE on the inner spatial-validation blocks over the fixed 0.01 grid; outer-test targets never enter this selection.

(26) $$w_{f}^{*}=\arg\;\min_{w\in \left\{0,0.01,\ldots ,1.00\right\}}\text{RMSE}\left(V_{f},{\hat{y}}^{F}\left(w\right)\right)$$

Equation 27 defines the final prediction ensemble as the arithmetic mean of fixed out-of-fold fusion predictions from seeds 17, 29, and 43.

(27) $${\hat{y}}_{e}^{E}=\frac{1}{\left|S\right|}\sum_{s\in S}{\hat{y}}_{e,s}^{F},\;S=\left\{17,29,43\right\}$$

For both the core and fusion models, per-seed predictions and three-seed ensembles are reported separately. Algorithm 1 summarizes block-exclusive training, inner validation, fusion, and ensemble construction.

Algorithm 1 **Block-exclusive spatial evaluation and validation-selected fusion for phenology-aware LCRA.**

Line:   Procedure**Inputs**:   Matched tensors X, M, C, G; targets Y for S−P and S−I; four outer folds; stage depths K = {1, 4}; seeds {17, 29, 43}.**Returns**:   Per-seed out-of-fold core/fusion member predictions, derived matched contrasts, core predictive variances, and three-seed prediction ensembles.1:   **for each** response task *q* in {S−P, S−I} **do**2:     **for each** seed *s* in {17, 29, 43} **do**3:       **for** outer fold *f* = 1, …, 4 **do**4:         *T_f_* ← complete held-out blocks; keep each matched triplet and all its events together; conceal *T_f_* targets during fitting and selection.5:         Rank remaining blocks by SHA-256(block_id | *s*+*f*); assign max[1, round(0.18 × blocks)] to *V_f_* and the remainder to *F_f_*.6:         Fit all normalizers on *F_f_* only; apply the stored transforms unchanged to *V_f_* and *T_f_*.7:         **for each** K in {1, 4}: optimize LCRA with **Equation 24**, retain the epoch minimizing member RMSE on *V_f_*, and store its predictions on *V_f_* and *T_f_*; **end for**.8:         Fit iTransformer under the identical *F_f_*/*V_f_*/*T_f_* split and target; store its predictions on *V_f_* and *T_f_*.9:         **for each** K: select w*_f,K_ on *V_f_* by **Equation 26**; form the **Equation 25** fusion on *T_f_* and store core/fusion predictions, contrasts, and core variance; **end for**.10:       **end for**; concatenate the four held-out partitions into one fixed out-of-fold prediction per member-event.11:     **end for**; average the three fixed seed predictions by **Equation 27**.12:   **end for Report**:   Retain both depths: K = 4 supplies maximum primary accuracy; K = 1 supplies the compact accuracy–cost Pareto configuration.

#### 2.3.2 Comparator hierarchy and controlled task adaptations

The primary comparison contained two conventional models and eight recent deep architectures. The conventional panel comprised a strong EVI-state-plus-season linear reference and extra trees (Geurts et al., 2006). The linear reference used pre-event EVI, impact EVI, their difference, and onset sine/cosine in outer-fold regression. Extra trees used 270 features: flattened temporal values and masks, climate-season and static tensors, and explicit pre/impact means. Its inner grid contained 800 trees, maximum-feature fractions 0.5/0.8/1.0, and minimum leaf sizes 1/2/4. A joint-output Ridge model provided an auxiliary linear diagnostic (Hoerl and Kennard, 1970).

The deep panel comprised PatchTST, TimesNet, iTransformer, TimeMixer, ModernTCN, TimeXer, WPMixer, and xPatch. Public source implementations supplied the forecasting cores, while fixed Qinling adapters repeated climate/static context over the eight dates and arranged each event as 8 × 72 channels for three-member prediction. PatchTST, TimesNet, iTransformer, and TimeMixer used their classification cores, ModernTCN retained its large-kernel classification path, TimeXer ran three member-specific forecasts with one endogenous EVI and 71 exogenous channels, and WPMixer/xPatch retained forecasting cores with a 72-to-3 task head. Masked temporal fields remained zero after fitting-partition normalization, while climate/static context was repeated at all eight positions. The adapters used sequence length 8, hidden size 64, feed-forward size 128, four heads, two encoder layers, one decoder layer, dropout 0.08, and a common optimization envelope while retaining architecture-specific operators and documented patch or downsampling settings. All architectures were evaluated under the common Qinling task protocol. PatchTST, TimesNet, iTransformer, TimeMixer, TimeXer, and WPMixer used THUML Time-Series-Library commit 4e938a1767106324dd753b2a44832bf870a0252e, ModernTCN used author-repository commit 56a9a2c018385cd5acef015378cae7f084d1b11c, and xPatch used author-repository commit d12eecaa11409109582f5e2ffdebcc2cffd47b3e. Adapter definitions, parameter counts, and training configurations are supplied in **Supplementary Table S1** and the accompanying model-source table. All neural models were trained and evaluated with CUDA on an NVIDIA GeForce RTX 4090 (THUML, 2026).

The comparison strategy combined broad single-seed evaluation with equal-seed replication of the strongest deep comparator. All eight deep models were evaluated at seed 17. iTransformer, the strongest deep model in that common-seed comparison, and extra trees were also evaluated at seeds 29 and 43 using the same three seeds, outer folds, and held-out member-events as LCRA. For each replicated model, we report both single-run mean ± sample standard deviation and the performance of an equally weighted three-seed prediction ensemble, separating architecture differences from averaging gains. The strong linear reference used a deterministic fourfold OOF fit. LCRA and its defined fusion were evaluated at seeds 17, 29, and 43. Architecture, input, and loss ablations used seed 17 and are reported as single-seed component analyses. The structured contrast-plus-NLL objective was transferred without changing data or folds to iTransformer, TimeXer, and TimesNet.

#### 2.3.3 Spatial evaluation, metrics, and hierarchical uncertainty

All model comparisons used four outer folds formed from complete, non-overlapping 50-km blocks (Roberts et al., 2017; Valavi et al., 2019; Wang et al., 2023). Every set and all its events remained in one fold, preventing an exposed member or control from appearing on opposite sides of a split. For each outer fold *f* and base seed *s*, blocks in the remaining training geography were ranked by the first 12 hexadecimal digits of SHA-256(block_id | s+f); the first max[1, round(0.18×ntraining blocks)] complete blocks formed the inner-validation partition. Inner validation governed checkpoint retention, conventional-model hyperparameters, and fusion weight. All continuous-input and target normalization statistics were fitted on the remaining inner-fitting partition. Concatenating the four outer-test partitions produced one out-of-fold prediction for every member-event. Stage depth was evaluated from the complete out-of-fold results of both independently trained configurations.

Equation 28 defines the coefficient of determination used as the primary accuracy metric; *y*_i_ and *ŷ*_i_ are the observed and predicted responses, and *ȳ* is the observed sample mean. RMSE and MAE were calculated in EVI units, and Pearson *r* was calculated using its standard sample definition.

(28) $$R^{2}=1-\frac{\sum_{i}{\left(y_{i}-{\hat{y}}_{i}\right)}^{2}}{\sum_{i}{\left(y_{i}-\bar{y}\right)}^{2}}$$

The same metrics were computed for 991 exposed-minus-control contrasts. Fold, block, set, onset month, year, role, and response-tail summaries described transfer stability. Macro metrics gave equal weight to sets or blocks rather than to their event counts.

Superiority against the task-specific reference models was resampled jointly over 32 spatial blocks and 281 shared onset dates. Bayesian and multinomial pigeonhole bootstraps each used 2,000 replicates and retained all three members of an event. Pairwise comparisons among trained models used 2,000 spatial-block bootstrap replicates, retaining every onset and matched member inside a sampled block. The primary superiority quantities were *R*^2^ gain, RMSE reduction, and MAE reduction. Observed regional contrasts were summarized under event-, set-, and block-equal estimands with block-by-onset dependence.

For the LCRA core, predictive variances from the three seeds were combined by the law of total variance, separating mean decoded variance from between-seed dispersion. Predictive dispersion was calibrated separately for each seed and outer fold using a positive scalar variance multiplier fitted on the original inner-validation blocks. The multiplier equals the mean squared standardized validation residual: the average squared prediction error divided by its predicted variance. This minimizes Gaussian NLL with the means held fixed. Each decoded variance was multiplied by this factor before adding the unchanged between-seed variance. Calibration preserved every point prediction and used no outer-test outcomes to estimate the scale. The inner-validation blocks also governed early stopping; this is validation-based variance scaling, with coverage assessed empirically on outer folds. Original and scaled Gaussian NLL, CRPS, interval width, and empirical 50%, 80%, 90%, and 95% coverage were evaluated overall and by spatial fold and matched role. Additional unscaled diagnostics summarize season and severity. Fusion uncertainty is reported as between-seed dispersion; predictive-interval metrics apply to the LCRA core’s variance decoder.

### 2.4 Vegetation interpretation and reliability

Application analyses used observed EVI responses and fixed out-of-fold predictions. Seasonal summaries used three prespecified onset groups: early season (May–June), midsummer (July–August), and late season (September). The principal canopy-state summaries divided pre-event mean EVI over all 2,973 member-events into quartiles. The monthly trajectory display used tertiles over the same full sample to retain stable support for low, intermediate, and high trajectories, whereas the primary canopy-state analysis used quartiles. Event severity combined simultaneous run length, maximum temperature exceedance, and integrated soil-moisture deficit. Each descriptor was standardized over the full member-event sample with population SD, the three *z* scores were averaged, and the resulting severity score was divided into quartiles. To separate severity-group response from calendar composition, member-level marginal means were estimated from outcome ~ severity quartile + onset month + year and standardized to the pooled month–year distribution of all 2,973 member-events. Uncertainty used 2,000 spatial-block × shared-onset Bayesian-pigeonhole replicates with weighted refitting and marginal standardization in every replicate.

Equation 29 residualizes predictor *x* and response *y* against the same design matrix *Z* containing an intercept plus month and year indicators. For either variable, the hatted alpha term is its ordinary least-squares coefficient vector.

(29) $$\tilde{x}=x-Z{\hat{\alpha }}_{x},\;\tilde{y}=y-Z{\hat{\alpha }}_{y},\;{\hat{\alpha }}_{v}={\text{arg min}}_{\alpha }{\left\|v-Z\alpha \right\|}_{2}^{2}$$

Equation 30 defines the adjusted raw-scale slope and rank-residual correlation. Average ranks are used for ties, and both rank vectors are residualized against the same *Z* before their Pearson correlation is computed.

(30) $${\hat{\beta }}_{x\to y}=\frac{\sum_{i}{\tilde{x}}_{i}{\tilde{y}}_{i}}{\sum_{i}{\tilde{x}}_{i}^{2}},\;\rho_{adj}=\text{Corr }\left(\tilde{\text{rank}\left(x\right)},\tilde{\text{rank}\left(y\right)}\right)$$

Intervals used 2,000 Bayesian-pigeonhole replicates with independent Dirichlet weights for the 32 blocks and 281 onset dates; their product weighted each member-event, retaining complete triplets. Multinomial pigeonhole resampling provided the corresponding sensitivity calculation. This organization preserves the spatial and shared-onset dependence while estimating vegetation gradients on the observed EVI scale.

The conditional mapped-landform analysis first reduced every complete set-event to an event-level contrast: the mapped-landform response minus the arithmetic mean of its two matched controls. Events then received equal weight. Season followed the prespecified May–June, July–August, and September groups; pre-event EVI quartiles and severity quartiles were assigned from the mapped-landform member so that each complete triplet entered one stratum. Reported strata required at least 20 events and three shared onsets; non-block strata additionally required three spatial blocks. The same 2,000-replicate spatial-block × shared-onset Bayesian-pigeonhole procedure supplied mean-contrast and negative-fraction intervals. Season (6), mapped-landform pre-state quartile (8), severity quartile (8), and displayed spatial-block (32) contrasts across S−P and S−I formed one prespecified family of 54 comparisons. Two-sided resampling tail probabilities used a plus-one correction and were adjusted once with the Benjamini–Hochberg procedure. Raw intervals and BH-adjusted *q*-values are reported together; all 32 block point estimates define the reported range, and 16 blocks met the display-support rule.

Reliability analyses related error and predictive uncertainty to geometry support, MODIS-cell support, role, and severity. Of 5,822 within-set event pairs, 232 (3.98%; 422/991 events across 77/80 sets) had overlapping nominal windows; the minimum onset separation was 40 days. All such pairs remained within one outer fold, with zero unit or spatial-block violations (**Supplementary Table S8**). Complete-set folds and spatial-block × shared-onset resampling retained this temporal clustering. A complete-row swap test evaluated row-order equivariance.

## 3 Results

### 3.1 Spatially generalizable canopy prediction

For the primary nominal composite-start-window task, the four held-out spatial folds returned one out-of-fold prediction for each of the 2,973 member responses. The compact *K* = 1 LCRA core averaged *R*^2^ = 0.8110 ± 0.0023 across three independently trained fits, and their prediction ensemble reached *R*^2^ = 0.8219, RMSE = 0.0662 EVI, and MAE = 0.0513 EVI. Against the EVI-state-plus-season linear reference (*R*^2^ = 0.7964), the paired member-level gain was Δ*R*^2^ = +0.0254 (95% spatial-block × shared-onset interval 0.0037–0.0532), positive in all three seeds and all four outer folds. Matched-contrast *R*^2^ was 0.3684 for LCRA and 0.3677 for the linear reference (Δ*R*^2^ = +0.0007; −0.0448 to 0.0425), locating the demonstrated advantage at member-level spatial transfer while retaining comparable contrast accuracy.

The EVI-state-plus-season linear model was the strongest comparator, reaching member *R*^2^ = 0.7964. Under equal three-seed replication, LCRA averaged *R*^2^ = 0.8110 ± 0.0023, compared with 0.7680 ± 0.0181 for iTransformer and 0.7811 ± 0.0003 for extra trees. Their equally weighted ensembles reached 0.8219, 0.7942, and 0.7815, respectively. LCRA exceeded iTransformer in each corresponding seed, and its ensemble retained a member-*R*^2^ margin of 0.0277 at the same ensemble size. This ordering aligns with the vegetation analysis: the linear reference directly represents current canopy state and seasonal position, the two dominant low-dimensional organizers of short-horizon EVI movement. Generic sequence architectures instead had to recover these signals from seven or eight irregular composite observations while transferring to unseen spatial blocks. LCRA combined those state and seasonal signals with dated temporal refinement, event climate, terrain, and matched-triplet structure. The compact fusion ensemble reached *R*^2^ = 0.8254 with RMSE = 0.0655 and MAE = 0.0507. These results describe the nominal-window member-level task (**Figure 4**; **Table 2**); complete-support results are reported separately below.

**Figure 4:**
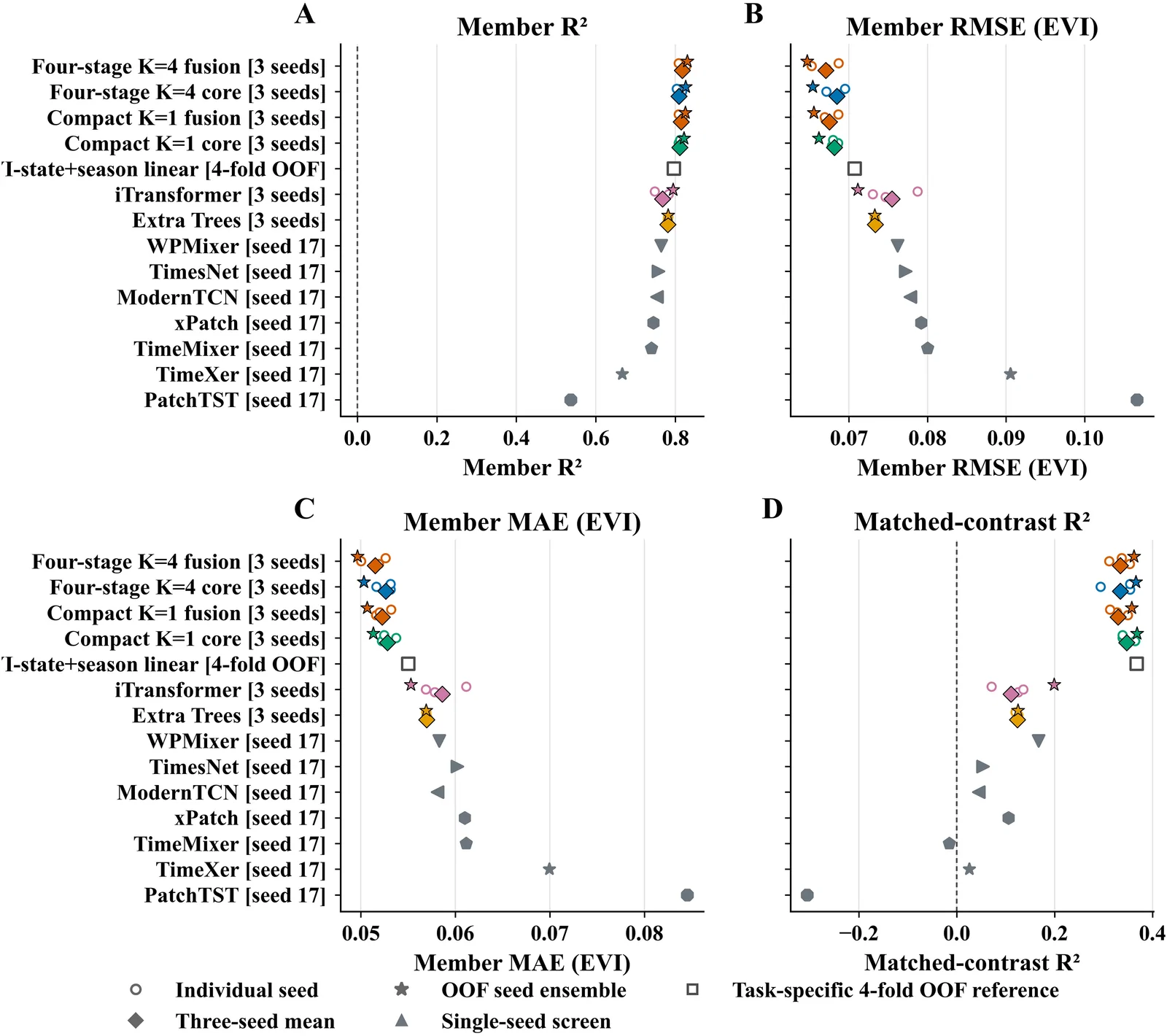
Spatial-CV benchmark under the common Qinling task protocol for S−P. **(A)** Member *R*^2^, **(B)** member RMSE, **(C)** member MAE, and **(D)** contrast *R*^2^. Horizontal point plots distinguish single-seed comparisons, replicated means, and out-of-fold prediction ensembles. Replicated LCRA, iTransformer, and extra trees use the same seeds (17, 29, and 43); ensemble comparisons average three aligned predictions per member-event. LCRA core and fusion are shown separately.

**Table 2:** Primary nominal-window S−P out-of-fold performance.

Comparator/model	Family	Evaluation result	Seeds	Member *R*^2^	RMSE	Contrast *R*^2^
EVI-state + season linear	Traditional linear	Deterministic 4-fold OOF reference	n/a	0.7964	0.0707	0.3677
Extra trees	Traditional nonlinear	Replicated mean ± SD	3	0.7811 ± 0.0003	0.0733 ± 0.0001	0.1236 ± 0.0031
Extra trees (prediction ensemble)	Traditional nonlinear	Three-seed averaged OOF prediction	3	0.7815	0.0733	0.1251
PatchTST	Deep time-series	Single-seed comparison	1	0.5370	0.1067	−0.3056
TimesNet	Deep time-series	Single-seed comparison	1	0.7574	0.0772	0.0536
iTransformer	Deep time-series	Single-seed comparison	1	0.7732	0.0747	0.1253
TimeMixer	Deep time-series	Single-seed comparison	1	0.7396	0.0800	−0.0153
ModernTCN	Deep time-series	Single-seed comparison	1	0.7536	0.0778	0.0447
TimeXer	Deep time-series	Single-seed comparison	1	0.6665	0.0905	0.0255
WPMixer	Deep time-series	Single-seed comparison	1	0.7639	0.0762	0.1670
xPatch	Deep time-series	Single-seed comparison	1	0.7448	0.0792	0.1057
iTransformer	Deep time-series	Replicated mean ± SD	3	0.7680 ± 0.0181	0.0755 ± 0.0029	0.1106 ± 0.0349
iTransformer (prediction ensemble)	Deep time-series	Three-seed averaged OOF prediction	3	0.7942	0.0711	0.1991
Compact K = 1 LCRA core (prediction ensemble)	Proposed core	Core prediction ensemble	3	0.8219	0.0662	0.3684
Compact K = 1 LCRA + iTransformer (prediction ensemble)	LCRA–iTransformer fusion	Fusion prediction ensemble	3	0.8254	0.0655	0.3573
Four-stage K = 4 LCRA core (prediction ensemble)	Proposed core	Core prediction ensemble	3	0.8261	0.0654	0.3658
Four-stage K = 4 LCRA + iTransformer (prediction ensemble)	LCRA–iTransformer fusion	Fusion prediction ensemble	3	0.8297	0.0647	0.3617

Replicated models use seeds 17, 29, and 43, the same four spatial folds, and the same 2,973 member-events; mean ± SD summarizes individual runs, whereas ensemble metrics are calculated after averaging three predictions. Single-seed rows are identified separately.

**Figure 5** displays spatially out-of-fold observed–predicted agreement for S−P in the top row and S−I in the bottom row, with one-stage fusion, one-stage LCRA core, and extra trees arranged in common columns. Across both tasks, the neural ensembles followed the 1:1 relation through the dense central canopy-response distribution and retained separation in the response tails, while the common 2 × 3 layout makes the post-impact difficulty shift directly visible without changing plot type. Event-level exposed-minus-control deviations also retained positive out-of-fold *R*^2^. The strong linear reference achieved contrast *R*^2^ = 0.3677, the one-stage core ensemble 0.3684, and fusion 0.3573; the core-minus-linear block × onset difference was 0.0007 (95% interval −0.0448 to 0.0425). LCRA’s principal measured advantage is therefore member-level prediction, while contrast accuracy is comparable to the state-plus-season reference. Within LCRA at seed 17, removing the relational refiner reduced contrast *R*^2^ from 0.3639 to 0.3503, isolating the component’s contribution inside the proposed architecture.

**Figure 5:**
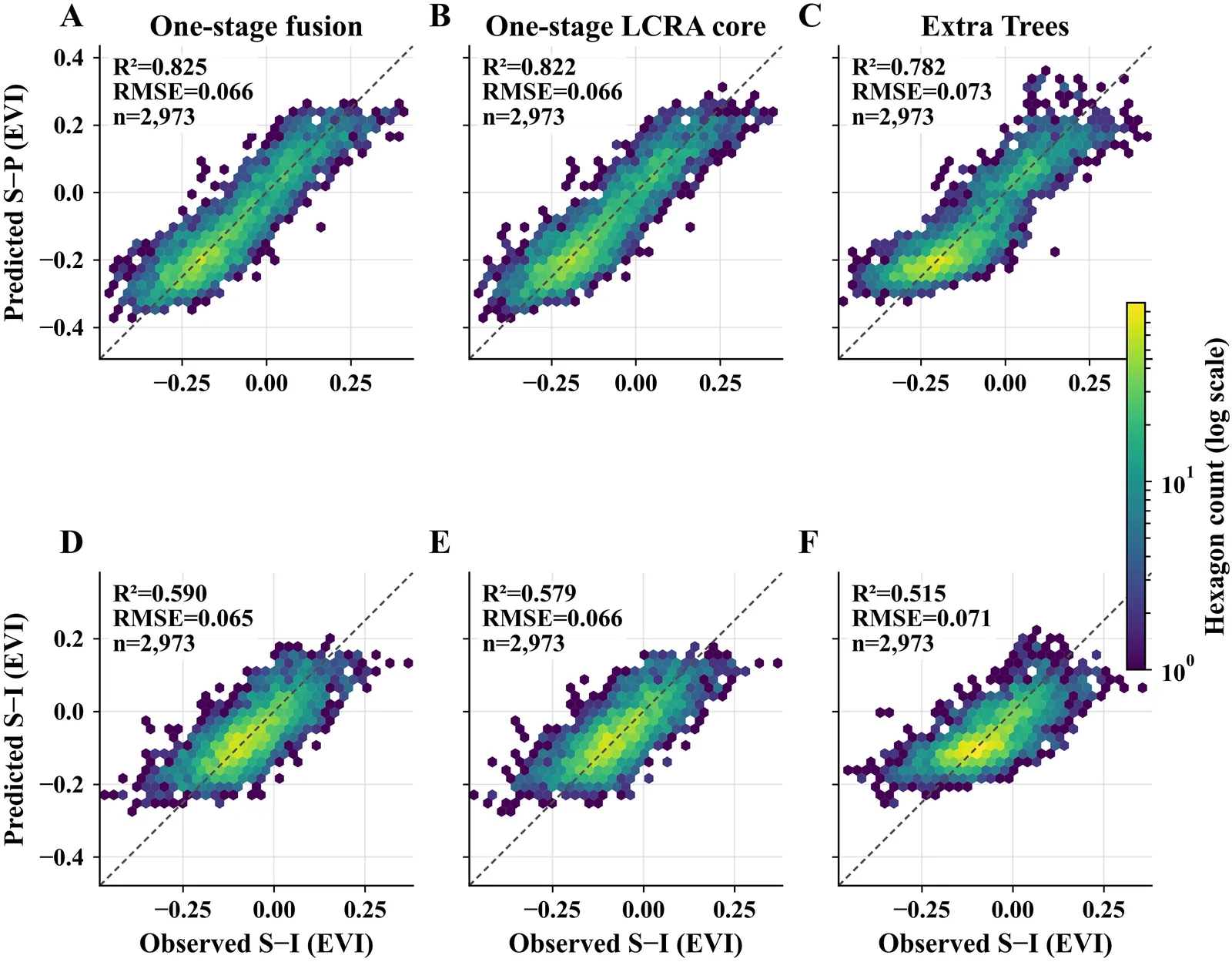
Spatially out-of-fold observed–predicted agreement. **(A)** S−P fusion, **(B)** S−P LCRA core, **(C)** S−P extra trees, **(D)** S−I fusion, **(E)** S−I LCRA core, and **(F)** S−I extra trees. All 2,973 targets use common axes, 1:1 lines, and log-count hexagons.

The performance ordering was replicated across three initializations. Core *R*^2^ ranged from 0.8084 to 0.8127, and fusion ranged from 0.8083 to 0.8179. The five replicated model layers—one-stage LCRA, four-stage LCRA, iTransformer, extra trees, and fusion—were compared seed by seed and fold by fold (**Figure 6**). LCRA maintained high accuracy in every held-out region, while the validation-selected fusion reduced the largest residual concentrations where the two neural experts made complementary errors. Fold-level dispersion was larger than seed-level dispersion, emphasizing that geography, not random initialization, was the dominant source of evaluation variation.

**Figure 6:**
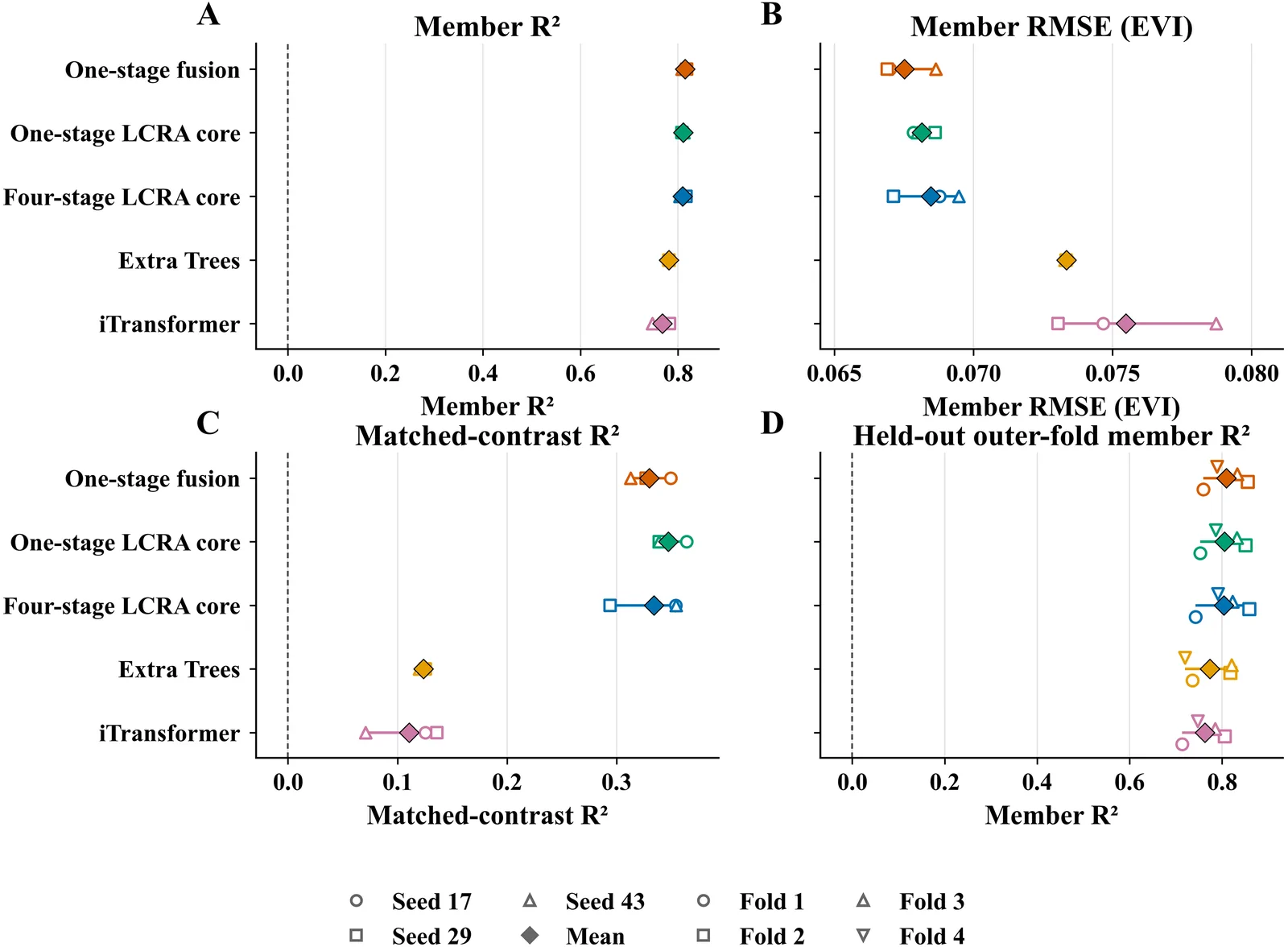
Replication and spatial-fold stability. **(A)** Member *R*^2^ across seeds, **(B)** member RMSE across seeds, **(C)** contrast *R*^2^ across seeds, and **(D)** member *R*^2^ by held-out fold. Model colors are fixed, and fold identity uses point shape.

### 3.2 What drives LCRA performance

The ablation analysis localized the member-level gain in climate–season and dated temporal information (**Figure 7**). Removing the six-variable event-climate plus two-variable season tensor reduced seed-17 member *R*^2^ from 0.8127 to 0.7009, while removing the time-aware refiner reduced it to 0.7878. Static landform descriptors supplied a smaller increment. The relational update had its clearest role in the matched endpoint: its removal changed contrast *R*^2^ from 0.3639 to 0.3503 while leaving member *R*^2^ essentially unchanged. LCRA therefore assigns complementary endpoint-specific roles: climate–season anchoring and dated temporal refinement drive the member-level margin, while the relational update concentrates its contribution in matched-contrast representation.

**Figure 7:**
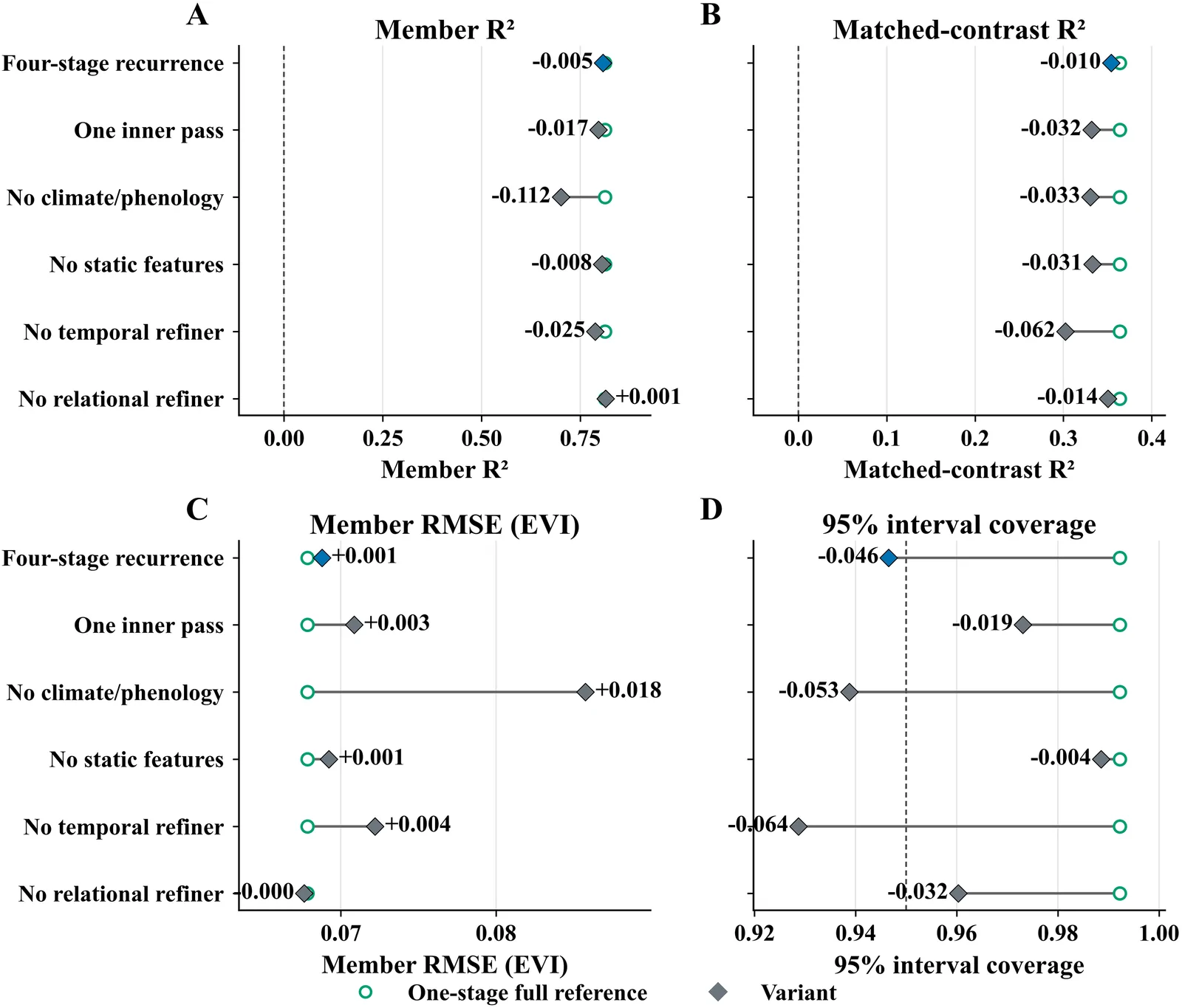
Architecture and input ablation at seed 17. **(A)** Member *R*^2^, **(B)** contrast *R*^2^, **(C)** member RMSE, and **(D)** 95% predictive-interval coverage. Each homogeneous dumbbell panel shows the change from full one-stage LCRA.

#### 3.2.1 Structured objective and loss transfer

The one-stage objective analysis separated member accuracy, matched-contrast supervision, and probabilistic output (**Figure 8**). Response-only training retained nearly all seed-17 member point accuracy (*R*^2^ = 0.8124 versus 0.8127 for the complete objective), showing that the member-response term alone supported strong prediction within the full architecture. Removing the contrast term changed matched-contrast *R*^2^ from 0.3639 to 0.3566. Removing Gaussian NLL preserved point accuracy at 0.8136 but removed the learned variance objective. The complete model produced input-dependent variances. Before calibration, empirical coverage exceeded each nominal level; validation-based variance scaling addressed this over-dispersion without changing the point predictions, as reported in the uncertainty analysis below.

**Figure 8:**
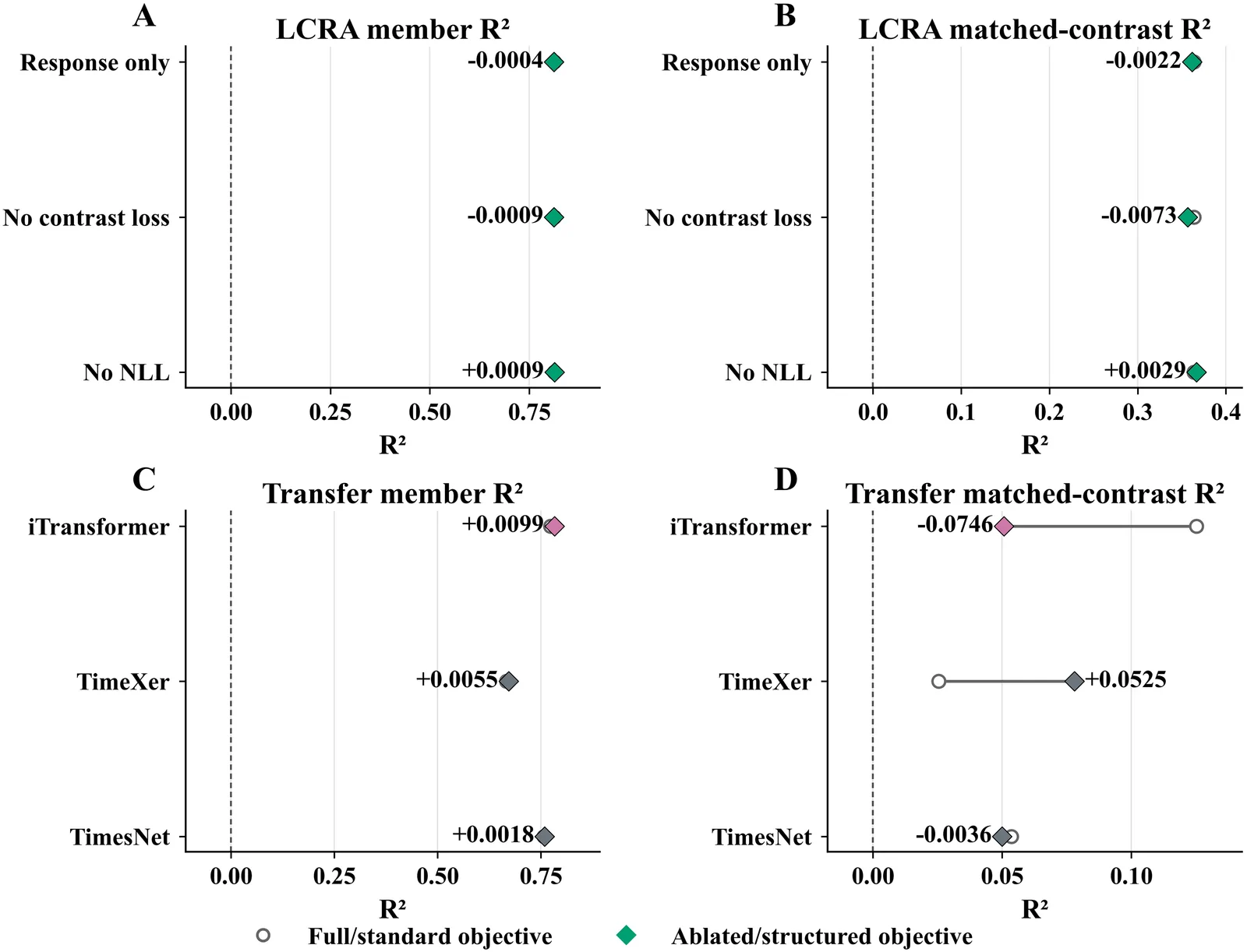
Structured-objective ablation and transfer at seed 17. **(A)** LCRA member *R*^2^ under loss ablation, **(B)** LCRA contrast *R*^2^ under loss ablation, **(C)** member *R*^2^ after transferring the structured objective to three deep comparators, and **(D)** the corresponding contrast *R*^2^.

At seed 17, transferring the structured objective increased member *R*^2^ by 0.0099 for iTransformer, 0.0055 for TimeXer, and 0.0018 for TimesNet. Contrast *R*^2^ changed by −0.0746, +0.0525, and −0.0036, respectively; TimeXer improved both endpoints, whereas iTransformer and TimesNet showed member gains with different contrast directions. The baseline adapters used global learned output log-variance parameters, whereas LCRA used an input-dependent heteroscedastic decoder. Loss transfer therefore provides single-seed evidence for consistent member gains and architecture-dependent contrast effects; LCRA and the baseline adapters use different variance parameterizations.

#### 3.2.2 Accuracy–cost frontier across stage depth

The complete out-of-fold comparison separates maximum primary accuracy from compact Pareto efficiency. Independently trained one-stage cores averaged 0.8110 ± 0.0023 across three seeds, compared with 0.8092 ± 0.0067 for the four-stage configuration, and their seed ensembles reached 0.8219 and 0.8261, respectively. Stage-wise decoding of the four-stage models showed that most predictive signal appeared after the first stage, followed by small seed-dependent corrections (**Supplementary Figure S5**). The four-stage fusion ensemble achieved the highest primary *R*^2^, 0.8297, whereas one-stage fusion reached 0.8254. The one-stage-minus-four-stage core difference was −0.0042 (95% block × onset interval −0.0166 to 0.0061); for S−I, the corresponding difference was +0.0132 (−0.0145 to 0.0476). Across three seeds, one-stage training reduced aggregate fourfold elapsed time by 59.4% and peak GPU memory by 53.1%. *K* = 4 therefore supplies the maximum-primary-accuracy configuration, while *K* = 1 is the compact Pareto configuration used for the main vegetation interpretation.

#### 3.2.3 Skill persists after removing the shared pre-event baseline

The S−I transition task removes the pre-event term shared by the primary target and impact anomaly. The EVI-state-plus-season linear reference fell to member *R*^2^ = 0.5141, while the one-stage LCRA ensemble retained member *R*^2^ = 0.5795 and matched-contrast *R*^2^ = 0.3488. The corresponding fusion ensemble reached member *R*^2^ = 0.5897 and contrast *R*^2^ = 0.3594. The strong linear contrast reference remained competitive at *R*^2^ = 0.3620, compared with 0.3488 for the core and 0.3594 for fusion. Substantial impact-to-subsequent movement remains predictable at member level from season, current EVI state, event climate, terrain, and matched context.

The paired dual-task display quantifies the difficulty shift (**Supplementary Figure S6**). Every model lost member-level *R*^2^ on S−I, while the core and fusion retained the leading member-prediction tier and positive matched-contrast skill. S−P delivered the strongest member-level predictive accuracy; S−I retained substantial skill for the impact-to-subsequent transition.

#### 3.2.4 Spatially clustered performance advantages

Spatial-block resampling retained all matched members and repeated events within each sampled block (**Supplementary Figure S7**). Against the strong linear reference, the nominal 80-set LCRA core ensemble gained Δ*R*^2^ = +0.0254 at member level (95% spatial-block × shared-onset interval 0.0037–0.0532), while fusion gained +0.0289 (0.0051–0.0622). One-stage LCRA also exceeded extra trees by Δ*R*^2^ = 0.0404 (0.0198–0.0695) for S−P and 0.0643 (0.0169–0.0969) for S−I; fusion increased these advantages to 0.0438 (0.0224–0.0757) and 0.0746 (0.0328–0.1021), respectively. For S−I, the fusion-minus-linear member gain was 0.0756 (0.0185–0.1311).

### 3.3 Complete-support and cohort sensitivities

Under the complete-support rule, the 80-set LCRA core ensemble retained member *R*^2^ = 0.780992 versus 0.776792 for the linear reference (Δ*R*^2^ = +0.004200; 95% interval −0.030354 to 0.035092). Matched-contrast *R*^2^ was 0.287542 and 0.328448, respectively (Δ*R*^2^ = −0.040906; −0.122590 to 0.023953). Complete temporal separation therefore retained strong absolute member-level prediction; the interval for its *R*^2^ difference included zero and did not establish superiority over the linear reference.

The same rule applied to all 90 eligible sets retained 20,266 of 26,464 input composite records and all 1,117 events, again with no boundary violation. The LCRA core ensemble reached member *R*^2^ = 0.798797 versus 0.774821 for the linear reference (Δ*R*^2^ = +0.023977; 95% interval −0.003737 to 0.054695). The relative *R*^2^ interval again included zero. In contrast, MAE decreased by 0.004129 EVI, with a positive interval of 0.000206–0.008744. All three LCRA seeds exceeded the linear member *R*^2^ point estimate, and all four folds reduced MAE. Matched-contrast Δ*R*^2^ was +0.009139 (−0.040458 to 0.056551). Thus, the complete-cohort analysis supports strong absolute accuracy and lower member-level MAE under non-overlapping support, while relative *R*^2^ and matched-contrast gains remain uncertain (**Supplementary Table S7**).

### 3.4 Vegetation response organization

The observed primary response averaged −0.0976 ± 0.1568 EVI across 2,973 member-events, whereas S−I averaged −0.0660 ± 0.1015. Season produced a strong sign reversal. For S−P, early May–June events averaged +0.0710 EVI (*n* = 993; 24.0% negative), while July–August and September averaged −0.1814 (*n* = 1,524; 96.7% negative) and −0.1849 (*n* = 456; 98.9% negative). The same seasonal ordering persisted for S−I: +0.0072, −0.1036, and −0.1000 EVI, with negative-response fractions of 45.1%, 90.5%, and 93.0%, respectively. This first application result establishes a seasonal transition from early-period greening relative to the comparison window to widespread midsummer and late-period decline.

Pre-event canopy state supplied a second, ordered axis (**Figure 9**). Mean S−P changed from +0.0260 in the lowest pre-state quartile to −0.0904, −0.1501, and −0.1762 across quartiles 2–4; the negative fraction increased from 46.6% to 68.1%, 84.9%, and 91.4%, respectively. S−I means were −0.0175, −0.0644, −0.0909, and −0.0914, with corresponding negative fractions of 57.7%, 76.0%, 85.7%, and 83.4%. Month- and year-adjusted pre-event EVI remained strongly negative for S−P (raw-scale coefficient −0.4759; adjusted Spearman *ρ* = −0.5185, 95% block × onset interval −0.6071 to −0.4347). Canopy state therefore organized response direction beyond coarse onset-month categories.

**Figure 9:**
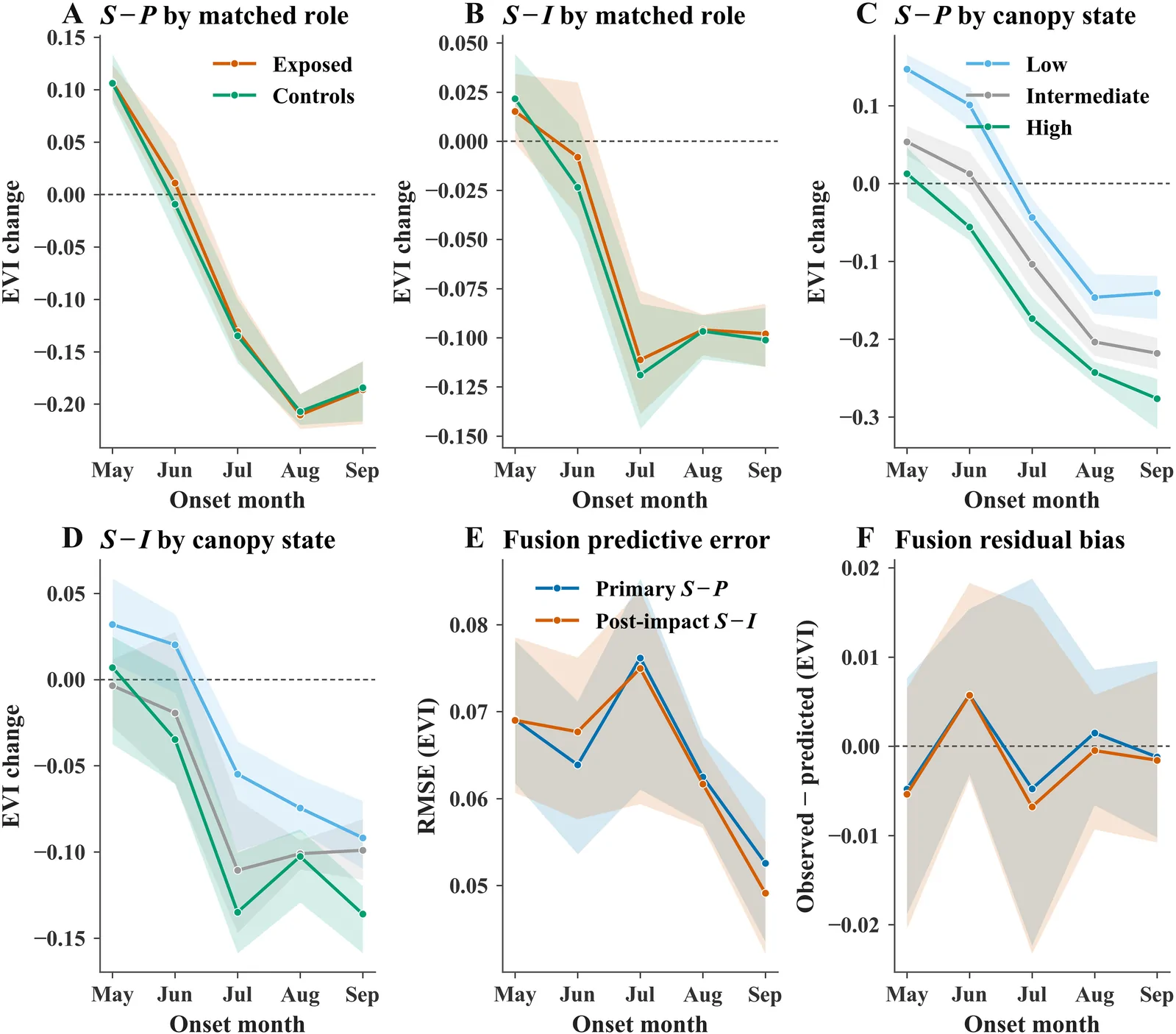
Monthly vegetation organization. **(A)** S−P by member role, **(B)** S−I by member role, **(C)** S−P by pre-event canopy state, **(D)** S−I by pre-event canopy state, **(E)** monthly fusion RMSE, and **(F)** monthly residual bias. Lines use the common monthly support. Shaded bands are 95% intervals from 2,000 spatial-block bootstrap resamples, retaining all events and matched members within each sampled block.

Calendar-adjusted associations separated the two response definitions more sharply. Impact anomaly was positively associated with S−P: the raw-scale coefficient was +0.5841 (95% block × onset interval 0.4869–0.7396), partial Pearson *r* = 0.5170, and adjusted Spearman *ρ* = 0.4975 (0.4122–0.5880). For S−I, the same impact-state predictor had coefficient −0.4159 (−0.5076 to −0.2725), partial Pearson *r* = −0.3950, and adjusted *ρ* = −0.3409 (−0.4499 to −0.1867). This sign reversal gives the paired endpoints distinct vegetation meanings: S−P expresses the subsequent canopy position relative to the pre-event state, whereas S−I expresses movement away from the impact-window state.

The pre-event state gradient persisted in the alternate endpoint after calendar adjustment. Its S−I coefficient was −0.1809 (−0.2880 to −0.0791), with adjusted *ρ* = −0.2005 (−0.3772 to −0.0515). Agreement among the quartile summaries, raw-scale coefficients, and rank-residual associations places initial canopy condition alongside season as a stable organizer of next-window EVI movement.

Impact anomaly was the dominant marginal correlate of S−P (Spearman *ρ* = 0.7668). Event duration (*ρ* = −0.1540) and integrated soil-moisture deficit (*ρ* = −0.1055) were negative in unadjusted summaries, but both changed direction after month and year were removed. Their adjusted raw-scale coefficients were +0.00328 EVI day−1 (Bayesian 95% block × onset interval 0.00052–0.00557) and +0.06063 per unit deficit (0.02007–0.09754); the adjusted rank correlations were small, and their intervals crossed zero. Heat intensity retained a positive adjusted association (coefficient +0.01167 EVI K−1, 0.00352–0.02006; adjusted Spearman *ρ* = 0.1272, 0.0030–0.2372). The analogous S−I coefficients were smaller—+0.00134 for duration, +0.00566 for heat exceedance, and +0.01882 for soil-moisture deficit—and all three coefficient intervals included zero. Event descriptors therefore contributed their clearest adjusted organization to the pre-state-relative endpoint.

Severity groups differed in calendar composition: early-season shares across Q1–Q4 were 19.4%, 38.0%, 48.2%, and 28.0%, whereas midsummer shares were 56.2%, 47.8%, 38.0%, and 63.1%, respectively (complete month/year composition in **Supplementary Table S9**). After standardization to the pooled month–year distribution, the S−P marginal means were −0.110704 (Q1; 95% interval −0.148964 to −0.068006), −0.117489 (Q2; −0.152226 to −0.073134), −0.077942 (Q3; −0.116266 to −0.035954), and −0.084397 (Q4; −0.120955 to −0.048205). The corresponding S−I means were −0.082957, −0.056532, −0.059509, and −0.065125. Both endpoints retained a non-monotonic ordering after calendar adjustment; the estimates distinguish severity contexts without defining a monotonic dose–response.

The adjusted terrain coefficients were smaller than the canopy-state coefficient but spatially coherent. For S−P, slope was negative (−0.00251 EVI degree−1; adjusted ρ = −0.1857, interval −0.3406 to −0.0479) and northness was negative (−0.03037; adjusted *ρ* = −0.1463, −0.2774 to −0.0266). S−I retained the aligned coefficients of −0.00215 for slope (−0.00368 to −0.00062) and −0.01880 for northness (−0.04246 to −0.00004). Eastness was weak for both endpoints, and its intervals crossed zero. Severity supplies a non-monotonic event axis, while canopy state, onset phase, slope, and northness provide the sharper vegetation structure.

### 3.5 Conditional mapped-landform behavior and reliability

Mapped landforms and controls had nearly identical regional response centers. For S−P, the control and exposed means were −0.0982 and −0.0964 EVI, with 73.6% and 71.1% negative responses; for S−I, they were −0.0668 and −0.0645, with 76.0% and 75.2% negative. The canonical event-equal exposed-minus-control mean was 0.00181 EVI for S−P (95% Bayesian block × onset interval −0.00504 to 0.00854) and 0.00234 for S−I (−0.00558 to 0.00977). Block-specific event-equal exposed-minus-control means ranged from −0.03554 to +0.03628 for S−P and from −0.02528 to +0.03219 for S−I, establishing bidirectional spatial variation around the near-zero regional center.

Event-equal weighting prevents the frequently observed sets from dominating, while complete triplets preserve the local matched reference.

Physical MODIS support was substantial across the 80 mapped geometries: 20–272 intersecting 250-m cells (median 28) and 12.53–214.06 Kish-effective cells (median 18.53). The controls had corresponding medians of 28 and 18.36 cells. The minimum event-window valid-cell fraction was 0.8261 for mapped landforms and 0.7037 for controls; no mapped-landform member-event and one of 1,982 control member-events fell below 0.80. Physical cell count showed little association with event-level |Δ| for either S−P (Spearman *ρ* = 0.0429; 95% interval −0.0601 to 0.1267) or S−I (*ρ* = 0.0419; −0.1067 to 0.1481), and support-quartile means showed no monotonic increase. Contrast magnitude was distributed across the observed support range rather than concentrated among low-support geometries (**Supplementary Table S8**).

**Figure 10** resolves conditional estimates by season, pre-event canopy state, and severity, while **Table 3** reports spatial blocks. Seasonal and severity contrasts remained close to the regional center. Across mapped-landform pre-state quartiles, S−P estimates ranged from +0.01968 in Q1 to −0.01979 in Q4; the Q4 raw 95% interval excluded zero (−0.03653 to −0.00319; BH *q* = 0.378), whereas S−I remained near zero. Spatial blocks contained the largest raw directional estimates, including B004_009 and B012_004 for both endpoints.

**Figure 10:**
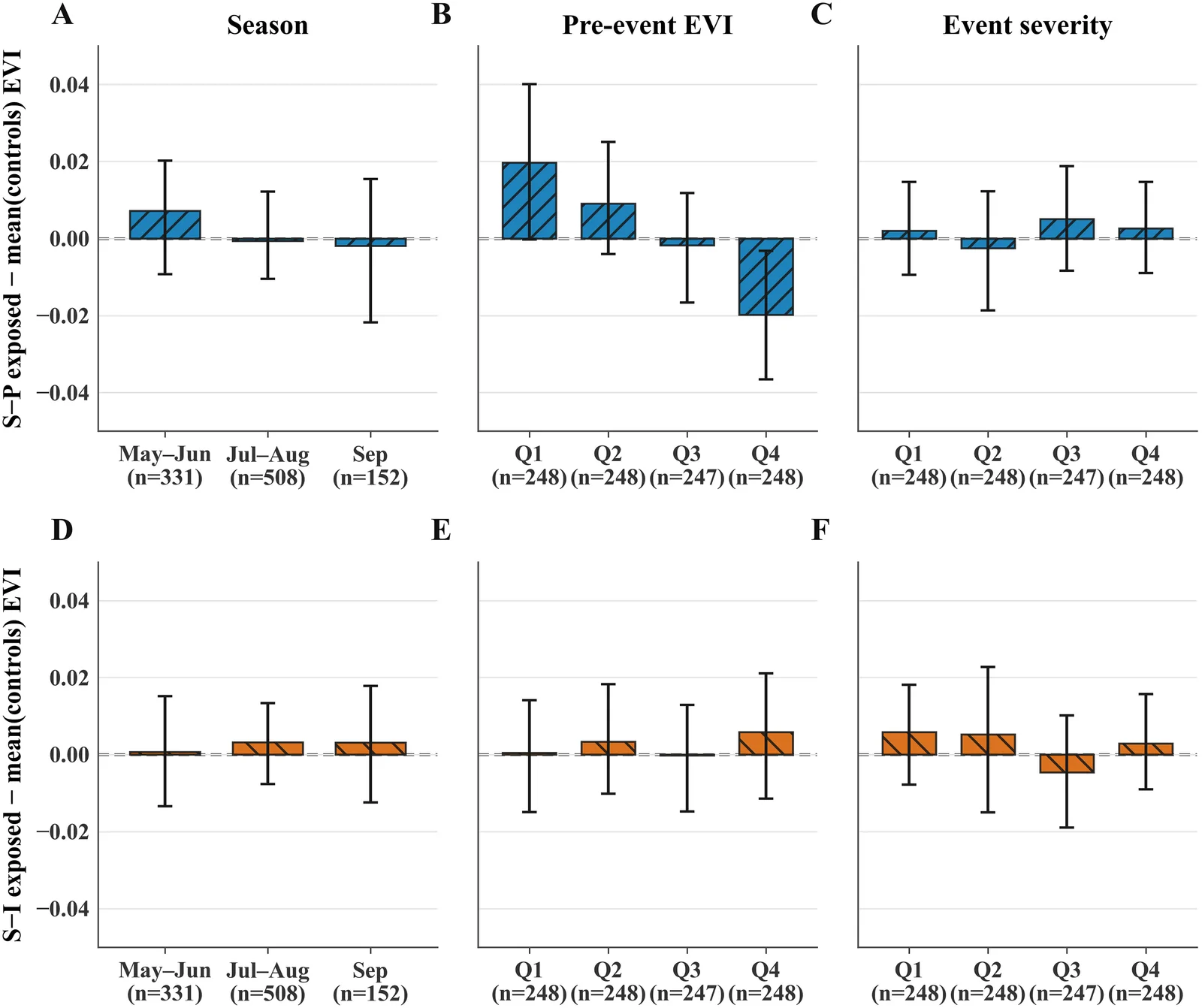
Conditional event-equal exposed-minus-control EVI departures. **(A)** S−P by season, **(B)** S−P by mapped-landform pre-event EVI, **(C)** S−P by severity, **(D)** S−I by season, **(E)** S−I by mapped-landform pre-event EVI, and **(F)** S−I by severity. Bars carry raw 95% intervals from 2,000 spatial-block × shared-onset Bayesian-pigeonhole replicates and report support counts. Benjamini–Hochberg *q*-values for the prespecified family of 54 conditional comparisons are reported in **Table 3** and **Supplementary Table S9**; no comparison had *q<*0.05.

**Table 3:** Spatial-block event-equal exposed-minus-control EVI departures.

Stratum	Task	*n*	Blocks/onsets	Mean contrast	Mean 95% B × O interval	BH *q*	Negative fraction	Negative-fraction 95% B × O interval
B003_003	S−P	181	1/53	0.0009	−0.0119 to 0.0121	0.973	50.3%	43.1% to 58.0%
B003_003	S−I	181	1/53	0.0043	−0.0127 to 0.0181	0.943	49.7%	42.0% to 58.6%
B003_002	S−P	144	1/48	0.0018	−0.0097 to 0.0125	0.944	48.6%	40.1% to 56.7%
B003_002	S−I	144	1/48	0.0015	−0.0121 to 0.0134	0.973	48.6%	42.2% to 55.3%
B003_004	S−P	66	1/47	−0.0005	−0.0154 to 0.0140	0.992	50.0%	38.7% to 60.7%
B003_004	S−I	66	1/47	0.0140	−0.0006 to 0.0278	0.439	48.5%	37.5% to 60.5%
B008_004	S−P	43	1/29	0.0119	−0.0064 to 0.0279	0.819	34.9%	23.5% to 47.5%
B008_004	S−I	43	1/29	0.0058	−0.0134 to 0.0246	0.943	46.5%	34.2% to 58.8%
B002_003	S−P	42	1/30	−0.0105	−0.0389 to 0.0169	0.943	57.1%	42.4% to 72.6%
B002_003	S−I	42	1/30	−0.0205	−0.0486 to 0.0063	0.746	54.8%	37.3% to 71.6%
B004_002	S−P	42	1/20	−0.0043	−0.0310 to 0.0238	0.943	54.8%	39.9% to 68.5%
B004_002	S−I	42	1/20	−0.0171	−0.0382 to 0.0011	0.506	59.5%	49.0% to 70.0%
B009_003	S−P	37	1/21	−0.0040	−0.0147 to 0.0080	0.943	51.4%	35.5% to 65.2%
B009_003	S−I	37	1/21	−0.0068	−0.0247 to 0.0078	0.943	54.1%	39.6% to 69.1%
B004_009	S−P	35	1/17	0.0124	0.0005 to 0.0215	0.432	28.6%	14.8% to 45.3%
B004_009	S−I	35	1/17	0.0092	0.0041 to 0.0142	0.054	37.1%	20.9% to 54.8%
B003_005	S−P	33	1/26	0.0043	−0.0163 to 0.0275	0.943	48.5%	32.9% to 62.5%
B003_005	S−I	33	1/26	0.0104	−0.0104 to 0.0284	0.889	45.5%	28.4% to 62.2%
B008_003	S−P	27	1/23	−0.0157	−0.0462 to 0.0074	0.826	48.1%	28.9% to 67.5%
B008_003	S−I	27	1/23	−0.0061	−0.0343 to 0.0245	0.943	59.3%	41.5% to 75.6%
B013_005	S−P	26	1/21	−0.0086	−0.0206 to 0.0018	0.702	57.7%	42.3% to 74.0%
B013_005	S−I	26	1/21	−0.0017	−0.0136 to 0.0101	0.944	53.8%	36.7% to 70.7%
B012_004	S−P	25	1/15	0.0211	0.0057 to 0.0427	0.081	36.0%	21.4% to 51.5%
B012_004	S−I	25	1/15	0.0188	0.0014 to 0.0396	0.418	40.0%	22.0% to 60.2%
B003_009	S−P	24	1/18	−0.0028	−0.0425 to 0.0343	0.986	41.7%	22.3% to 62.6%
B003_009	S−I	24	1/18	0.0124	−0.0217 to 0.0474	0.943	33.3%	15.2% to 53.2%
B009_002	S−P	24	1/19	0.0003	−0.0192 to 0.0220	0.992	58.3%	40.9% to 73.6%
B009_002	S−I	24	1/19	0.0098	−0.0129 to 0.0356	0.943	50.0%	29.1% to 71.0%
B014_001	S−P	22	1/13	0.0048	−0.0136 to 0.0231	0.943	59.1%	36.6% to 78.8%
B014_001	S−I	22	1/13	0.0146	−0.0069 to 0.0371	0.844	54.5%	33.3% to 77.6%
B012_006	S−P	20	1/15	0.0078	−0.0091 to 0.0230	0.943	35.0%	15.7% to 59.1%
B012_006	S−I	20	1/15	−0.0058	−0.0223 to 0.0098	0.943	60.0%	36.0% to 81.6%

Raw intervals are 2,000-replicate spatial-block × shared-onset Bayesian-pigeonhole intervals. BH *q*-values adjust the prespecified family of 54 conditional comparisons spanning season, pre-event canopy state, severity, and spatial block; all comparisons are retained in **Supplementary Table S9**.

Across the prespecified family of 54 conditional comparisons, five raw 95% intervals excluded zero, whereas no BH-adjusted *q*-value was below 0.05 (minimum *q* = 0.053973). The complete estimate set maps the concentration, magnitude, and direction of canopy-state and spatial variation and defines testable ecological hypotheses for independent data (**Table 3**; **Supplementary Table S9**).

Validation-based variance scaling improved predictive dispersion while retaining the original point predictions. For the nominal-window S−P task, empirical coverage at nominal 50%, 80%, 90%, and 95% levels changed from 71.64%, 94.08%, 98.42%, and 99.43% to 51.23%, 78.37%, 87.55%, and 92.94%, respectively. Mean 95% interval width decreased from 0.3977 to 0.2442 EVI (38.6%), Gaussian NLL improved from −1.1599 to −1.2925, and CRPS improved from 0.03921 to 0.03683. Coverage error decreased at all four levels. The calibrated 95% interval retained 2.06 percentage points of pooled undercoverage, with empirical coverage ranging from 90.65% to 96.92% across outer folds; NLL and CRPS improved in every outer fold.

The same validation-based procedure improved dispersion in the S−I task: 95% coverage changed from 97.78% to 92.70%, mean interval width decreased from 0.3207 to 0.2426 EVI, NLL improved from −1.2583 to −1.2759, and CRPS improved from 0.03728 to 0.03665. Coverage error decreased at all four reported levels for this task as well. **Supplementary Figure S1** and **Supplementary Table S2** compare original and scaled uncertainty across tasks and matched roles. The nominal-window calibration results are distinct from the complete-support point-prediction sensitivities. Residual plots localize shrinkage at response extremes and months with the largest spatial variation (**Supplementary Figure S2**), while **Supplementary Figure S3** quantifies the reduction in impact-anomaly coupling from S−P to S−I. Computational-budget diagnostics for stage depth are provided in **Supplementary Figure S4**, and complementary persistence, phenology, state-plus-season, and algebraic-identity diagnostics for both response tasks are reported in **Supplementary Table S3**.

Landform-scale mapping exposed spatial structure that was obscured by the near-zero regional contrast (**Figure 11**). Across 991 events, the 80 mapped units contributed 9–19 events each (median 12). Observed event-equal S−P contrasts ranged from −0.0447 to +0.0601 EVI, whereas the three-seed fusion out-of-fold contrasts ranged from −0.0643 to +0.0634 EVI. Prediction recovered the observed unit ordering (Spearman ρ = 0.5297), and observed and predicted contrasts had the same sign in 54 of 80 units. The mapped signal therefore consisted of spatially alternating positive and negative canopy departures that were partially retained in the mapped out-of-fold unit predictions.

**Figure 11:**
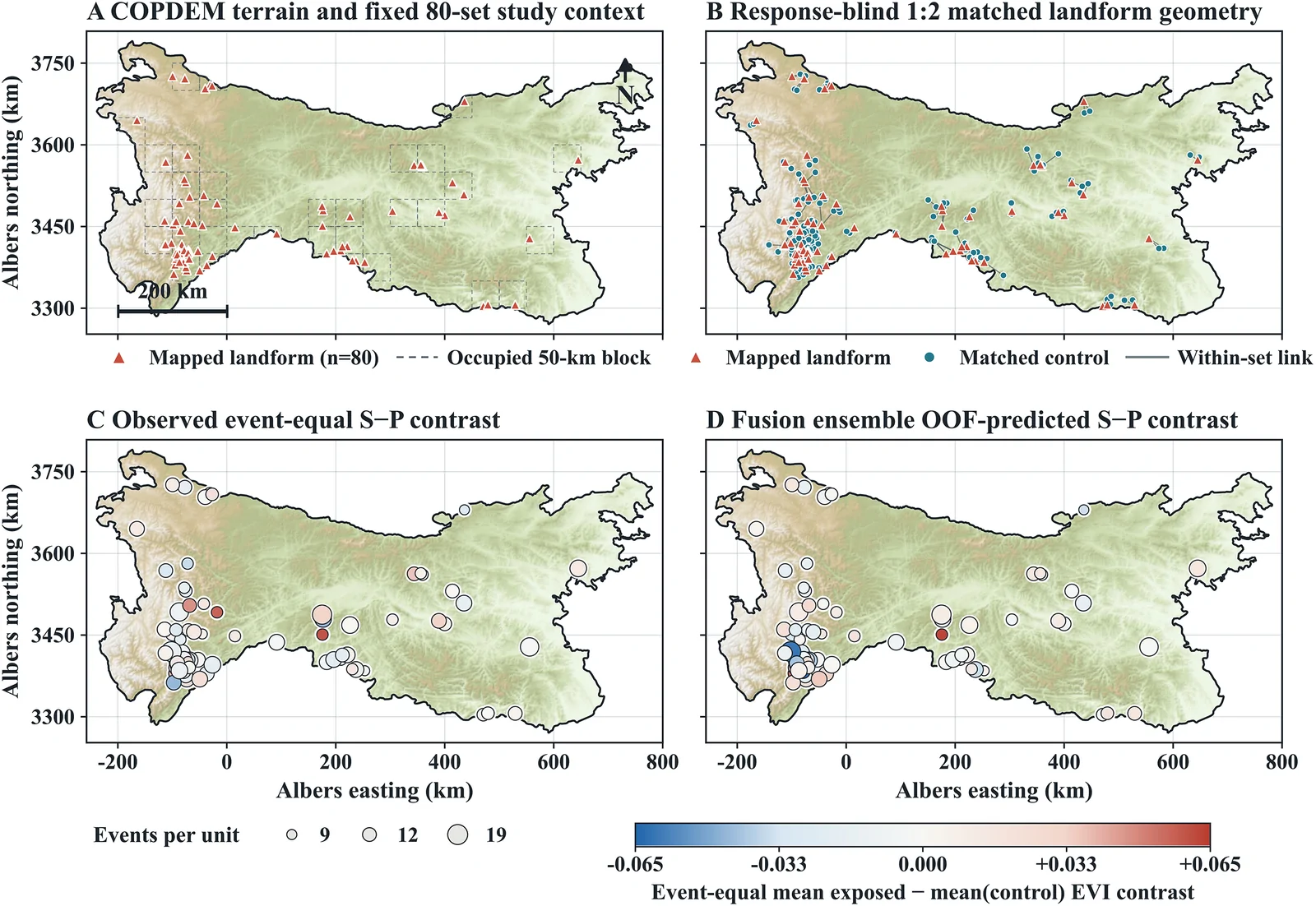
Spatial organization of the 80-set matched-landform design and event-centered S−P canopy departures across the Qinling region. **(A)** COP-DEM terrain context, Qinling boundary, 32 occupied 50-km blocks, and the 80 mapped landforms. **(B)** Response-blind 1:2 geometry linking the 80 mapped landforms to 160 same-event matched non-landslide controls. **(C)** Event-equal observed exposed-minus-control S−P contrast within each mapped landform. **(D)** Corresponding three-seed fusion out-of-fold contrast. **(C, D)** use the same zero-centered scale; symbol area represents 9–19 events per landform. Values are shown at true landform centroids, while COP-DEM provides terrain context.

Phenological phase further reorganized this landform-scale signal (**Figure 12**). All 80 units contributed to both displayed phases, with 331 events in May–June and 508 in July–August. The unweighted mean across the 80 unit-specific S−P estimates decreased from +0.00766 to +0.00194 EVI; 42 units (52.5%) changed sign, and early-to-midsummer rank association was negative (Spearman ρ = −0.2095). For S−I, the corresponding mean increased from +0.00097 to +0.00388 EVI, while 34 units (42.5%) changed sign and cross-phase rank association remained weak (ρ = 0.1003). Small regional centers thus coexisted with strong seasonal relocation and sign reversal of matched canopy departures.

**Figure 12:**
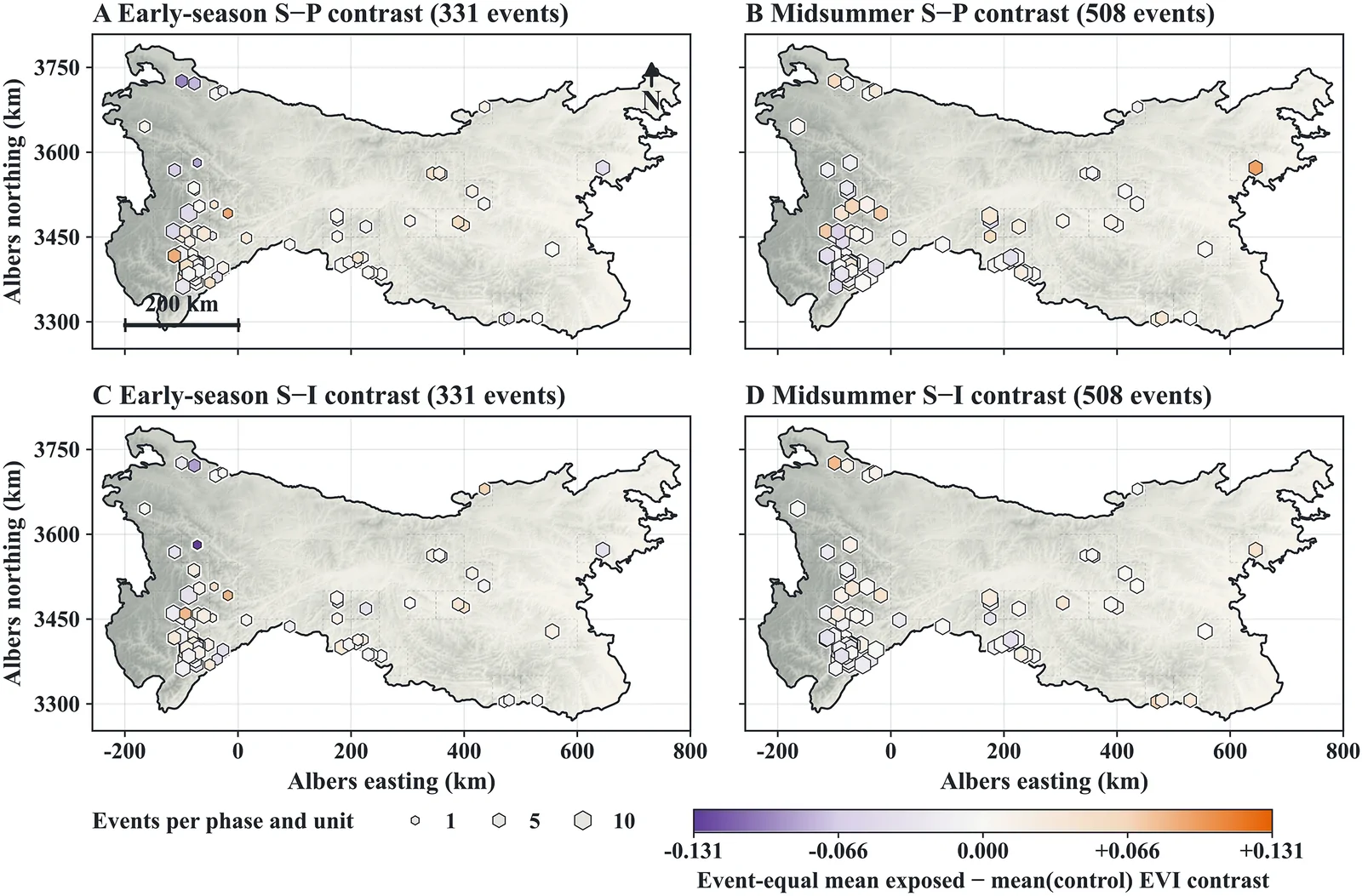
Seasonal reorganization of event-centered canopy departures across the 80 mapped Qinling landforms. **(A)** May–June S−P contrast based on 331 events, **(B)** July–August S−P contrast based on 508 events, **(C)** May–June S−I contrast, and **(D)** July–August S−I contrast. Each value is the event-equal mean exposed response minus the mean of its two controls within a landform and phase. All panels use the same zero-centered scale; the hexagon area denotes support. Values are located at true landform centroids over COP-DEM relief.

## 4 Discussion

### 4.1 Spatially transferred prediction under complementary temporal specifications

LCRA combines strong member-level spatial transfer with a joint representation of event-aligned canopy responses and matched-landform contrasts. In the primary nominal-window task, the compact core ensemble reached *R*^2^ = 0.8219 versus 0.7964 for the EVI-state-plus-season reference (Δ*R*^2^ = +0.0254; 95% spatial-block × shared-onset interval 0.0037–0.0532), and validation-selected fusion reached *R*^2^ = 0.8254 (Δ*R*^2^ = +0.0289; 0.0051–0.0622). These member-level gains apply to the composite-start-defined monitoring task. Under complete input–target support separation, absolute member R^2^ remained 0.7810–0.7988; relative R^2^ intervals included zero, while the full-cohort MAE reduction was supported. This distinction separates evidence for accurate canopy prediction from evidence for outperforming a task-aligned reference.

The comparator ordering further clarifies the task. The linear reference directly supplies current EVI state and seasonal phase, whereas generic deep forecasting architectures must infer them from a short irregular sequence. Its strength therefore reflects task alignment. LCRA adds dated temporal refinement, event climate, static landform context, and matched-triplet representation to those dominant signals. Removing the climate–season tensor produced the largest member-level loss, removing the time-aware refiner produced the next largest loss, and the relational update contributed most clearly to matched-contrast representation. Under the common adapters and block-exclusive nominal-window evaluation, the one-stage seed-17 LCRA model exceeded all eight recent deep comparators. The equal-seed comparison with the strongest deep comparator retained that ordering: three-seed mean *R*^2^ was 0.8110 for LCRA versus 0.7680 for iTransformer, and equal-size ensemble *R*^2^ was 0.8219 versus 0.7942. The ordering is therefore present in both individual-run and prediction-ensemble comparisons.

The objective experiments sharpen this interpretation. At seed 17, transferring the complete structured objective yielded member-level gains in iTransformer, TimeXer, and TimesNet; the experiment evaluates the objective as a whole, and contrast changes were architecture-dependent, with a joint gain in TimeXer. Within LCRA, the contrast term supported the matched endpoint and Gaussian NLL supplied the learned uncertainty output. The transfer result therefore identifies the complete structured objective as a transferable member-prediction component, with contrast effects that depend on architecture.

Stage depth separates the highest-accuracy and compact settings. *K* = 4 produced the maximum primary fusion *R*^2^ of 0.8297; *K* = 1 was 0.0043 lower while reducing aggregate fourfold training time across three seeds by 59.4% and peak GPU memory by 53.1%. Most four-stage signal appeared after the first decoded stage. *K* = 1 therefore defines the compact Pareto configuration for repeated spatial analysis, whereas *K* = 4 defines the maximum-primary-accuracy configuration.

Core and fusion provide distinct endpoint-specific outputs. Fusion combines LCRA’s task-structured representation with iTransformer’s variable-token representation and supplies the strongest compact member accuracy. The LCRA core retains the stronger compact matched-contrast output and its input-dependent variance decoder. For S−P contrast, core *R*^2^ = 0.3684 was comparable with the linear reference at 0.3677; the core-minus-linear difference was 0.0007 with a 95% interval of −0.0448 to 0.0425. The principal measured gain is member prediction, while the matched output preserves a complementary event-level landform signal.

### 4.2 Phenological phase and canopy state organize coherent greenness dynamics

The vegetation results reveal a pronounced season-conditioned sign reversal. Early May–June S−P was positive on average (+0.0710 EVI), whereas July–August and September were strongly negative (−0.1814 and −0.1849), with more than 96% of observations below zero in the latter periods. S−I preserved the same seasonal ordering at smaller magnitude. The 32-day subsequent window therefore moves through distinct portions of the annual greenness trajectory: early onsets are commonly followed by seasonal canopy rise, whereas midsummer and September windows more often span plateau-to-senescence or declining segments. Phenological phase is consequently the first organizer of the sign and scale of short-horizon EVI movement during the sampled compound-event periods. This phenology-centered ordering agrees with satellite evidence that vegetation-index phenology varies between years and can retain drought legacies in subsequent leaf dynamics (Gonçalves et al., 2020; Kato et al., 2021).

Pre-event canopy state supplies a second, ordered axis. Mean S−P changed from +0.0260 in the lowest pre-state quartile to −0.1762 in the highest, accompanied by an almost twofold increase in the negative-response fraction. S−I retained this gradient after removal of the shared pre-term, although the upper two quartiles flattened. The ordered pattern is consistent with biological state continuity, EVI’s bounded range, and difference-target geometry: high initial greenness leaves less upward headroom, high-canopy months more often precede seasonal decline, and subtracting a high reference strengthens ceiling and regression-to-the-mean structure. Persistence in S−I and after month/year adjustment shows that the gradient extends beyond one shared subtraction term.

The paired endpoints separate two useful views of this trajectory. S−P positions the subsequent canopy window relative to recent pre-event conditions and delivers the highest prediction accuracy. S−I measures movement from the impact window to the subsequent window and retains member *R*^2^ = 0.5795 for the core ensemble and 0.5897 for fusion. Their opposite calendar-adjusted associations with impact anomaly follow directly from their reference windows: S−P shares the pre-event anchor with the impact anomaly, while S−I subtracts the impact-window state. Together they distinguish a high-signal pre-state-relative monitoring quantity from an impact-to-subsequent transition.

Calendar-standardized severity-quartile means remained negative and non-monotonic for both endpoints, with lower S−P means in Q1–Q2 than Q3–Q4 but no ordered dose–response. Severity therefore contributes a secondary stratification around the stronger phenological, canopy-state, slope, and northness organization. The resulting vegetation hierarchy is quantitative: onset phase was the dominant organizer of response direction, initial greenness ordered attainable movement, and event descriptors and terrain were associated with secondary variation around those axes.

### 4.3 Conditional landform contrasts reveal localized ecological structure

The thematic maps resolve the spatial arrangement and seasonal reorganization of conditional estimates around a near-zero regional center (**Figures 11**, **12**). At landform scale, observed S−P departures spanned −0.0447 to +0.0601 EVI; mapped out-of-fold fusion predictions partially recovered their ordering (Spearman *ρ* = 0.5297) and matched their sign in 54 of 80 units. From May–June to July–August, 42 units reversed S−P sign and 34 reversed S−I sign. None of the 54 conditional comparisons met BH *q<*0.05; their mapped directions define ecological hypotheses and candidate strata for subsequent field or independent-data evaluation. The spatially alternating estimates align with established evidence that landslide terrain supports heterogeneous vegetation succession and ecosystem chronosequences (Yunus et al., 2020; Rasigraf and Wagner, 2022).

Positive contrast-prediction *R*^2^ shows that part of the event-level heterogeneity is learnable, while the strong linear contrast reference identifies the structure already carried by phenological state. In the nominal-window task, LCRA adds higher member-level accuracy and a joint matched output for the same event; the regional mean remains near zero and contrast accuracy remains comparable with the linear reference. Response-blind matching achieved complete 12-variable balance (all |SMD|< 0.20), complete-triplet folds preserved local comparisons, and the complete-row swap difference below 10−7 confirmed row-order equivariance. Physical MODIS cell support was nearly unrelated to |Δ| and showed no monotonic support gradient, placing the mapped contrast structure across the observed support range.

Predictive uncertainty complements the member and matched outputs when its scale is evaluated against held-out observations. Inner-validation variance scaling reduced the original over-dispersion, improved NLL and CRPS, and shortened primary-task 95% intervals by 38.6%, while leaving point accuracy unchanged. Empirical 95% coverage of 92.9% for S−P and 92.7% for S−I describes the resulting calibration more precisely than the nominal level alone. Variation across spatial folds shows spatial heterogeneity in predictive-interval reliability. Each core prediction can thus be read through three coordinated quantities: member-level canopy movement, an exposed-minus-control departure among same-event matched locations, and a validation-scaled predictive interval with measured coverage. Fusion uncertainty remains a separate summary of between-seed dispersion.

### 4.4 Transferable evidence for Qinling vegetation monitoring

The study targets event-aligned short-horizon EVI prediction and matched-landform comparison within the sampled Qinling period. MCD12Q1 land cover from 2020 supplied one response-blind matching anchor. Land-cover similarity denotes the 2020 landscape context; class stability and pairwise agreement across event years were not assessed. Event-aligned MODIS EVI and ERA5-Land supplied time-varying canopy and climate information, without establishing annual land-cover equivalence. S−P and S−I quantify observed window-to-window canopy transitions, and the matched contrast quantifies departures between contemporary mapped landforms and same-event controls. Because the design contains no non-event temporal counterfactual, these responses describe event-aligned canopy trajectories and associations with canopy state and season, not causal effects of hot–dry events.

The complete-support sensitivity required the entire 16-day support of each retained input composite to end by day +31 before the target began on day +32. The LCRA core ensemble achieved member *R*^2^ = 0.7810 in 80 sets and 0.7988 across all 90 eligible sets. Its corresponding *R*^2^ differences from the linear reference were +0.0042 (95% interval −0.0304 to 0.0351) and +0.0240 (−0.0037 to 0.0547). These analyses establish high absolute member-level accuracy under separated temporal support, with uncertain relative *R*^2^ superiority. The complete 90-set analysis additionally supports a member-level MAE reduction of 0.0041 EVI (0.0002–0.0087), with lower MAE in every outer fold. Matched-contrast differences remained uncertain under both cohort specifications.

The evaluation combines 991 events and 2,973 member responses from 80 matched sets in 32 occupied blocks. Chronologically overlapping windows comprised 232 of 5,822 within-set event pairs (3.98%); every pair remained within one outer fold. Complete-set block-exclusive folds and spatial-block × shared-onset resampling carry this temporal clustering into evaluation and interval estimation. Geographic transfer was demonstrated across unseen Qinling blocks, while other mountain systems, longer horizons, and denser temporal sensors define direct tests of transport beyond the present setting.

## 5 Conclusions

LCRA predicts event-aligned canopy variation across unseen Qinling blocks by combining climate–season context, dated temporal refinement, and matched-landform representation. In the nominal-window analysis, the compact core and fusion ensembles reached member *R*^2^ = 0.8219 and 0.8254 and exceeded the EVI-state-plus-season reference. Complete temporal-support separation retained member *R*^2^ = 0.7810–0.7988, while relative *R*^2^ gains remained uncertain; the full 90-set cohort supported lower member-level MAE. Equal three-seed comparison with iTransformer retained LCRA’s nominal-window member advantage, and validation-based variance scaling improved probabilistic scores and interval sharpness without changing point predictions. Across the vegetation application, seasonal sign reversal and pre-event canopy state organized the observed trajectories, with terrain and non-monotonic severity supplying additional context. Matched contrasts represented bidirectional spatial variation around a near-zero regional center. These findings link accurate spatial canopy prediction to a phenology-centered description of observed event-aligned vegetation dynamics.

## Data Availability

The raw data supporting the conclusions of this article will be made available by the authors, without undue reservation.
