# Supplementary material for: LCRA predicts event-aligned canopy responses across unseen Qinling landscapes
Source: Front Plant Sci. 2026 Oct 28;17:1955922. doi: 10.3389/fpls.2026.1955922 (PMC13752087; doi:10.3389/fpls.2026.1955922)
Supplement: Supplementary file 1 [file Table1.docx]

Supplementary material
Supplementary figures

Supplementary Figure S1. Predictive-interval reliability. (A) Primary S−P coverage overall, (B) primary S−P coverage by member role, (C) post-impact S−I coverage overall, and (D) post-impact S−I coverage by member role. Shading denotes 95% spatial-block bootstrap intervals; core variance combines decoded within-seed variance and between-seed mean dispersion.

Supplementary Figure S2. Residual structure of out-of-fold fusion predictions. (A) S−P residual versus fitted value, (B) S−I residual versus fitted value, (C) S−P residual versus observed response, (D) S−I residual versus observed response, (E) S−P residual versus impact anomaly, (F) S−I residual versus impact anomaly, (G) S−P residual versus onset timing, and (H) S−I residual versus onset timing. Black traces are equal-count-bin medians.

Supplementary Figure S3. Shared-baseline coupling. (A) Impact anomaly versus observed S−P, (B) impact anomaly versus observed S−I, (C) impact anomaly versus S−P residual, and (D) impact anomaly versus S−I residual. Annotations report Spearman association and spatial-block bootstrap intervals.

Supplementary Figure S4. Computational budget under the common primary-task protocol. (A) Trainable parameters, (B) fold training time, (C) best epoch, and (D) peak allocated GPU memory. All networks were evaluated at seed 17; each point represents one outer fold and diamonds show across-fold centers. Fusion reuses trained predictors and therefore has no additional network-training cost.

Supplementary Figure S5. Stage-depth accuracy–cost frontier. (A) Member R², (B) member RMSE, (C) matched-contrast R², and (D) matched-contrast RMSE for independently trained K = 1 models and stage-wise K = 4 predictions.

Supplementary Figure S6. Paired S−P/S−I performance under identical folds and seeds. (A) Member R², (B) member RMSE, (C) member MAE, and (D) contrast R². Each homogeneous paired-point panel links the two response definitions for the same model.

Supplementary Figure S7. Spatially paired ensemble advantages. (A) Primary-task ΔR², (B) primary-task RMSE reduction, (C) post-impact ΔR², and (D) post-impact RMSE reduction. Forest plots show full-sample differences and 2,000 complete-block bootstrap intervals; positive RMSE reduction favors the first model.
Supplementary tables
Supplementary Table S1. Training cost and resource use of one-stage and four-stage LCRA across three seeds and of eight deep comparators evaluated at seed 17. Elapsed time is summed over the four outer folds, peak memory is the maximum fold allocation, and all GPU measurements were recorded on an NVIDIA GeForce RTX 4090.
Model and seed	Analysis role	Parameters	Mean epochs	Mean best epoch	Total four-fold time (s)	Peak GPU memory (MB)	Member R²
LCRA core, K = 1 (seed 17)	Replicated compact LCRA core	141,603	54.5	9.5	68.0	36.2	0.8127
LCRA core, K = 1 (seed 29)	Replicated compact LCRA core	141,603	59.5	14.5	76.2	36.2	0.8084
LCRA core, K = 1 (seed 43)	Replicated compact LCRA core	141,603	56.2	11.2	76.8	36.2	0.8120
LCRA core, K = 4 (seed 17)	Replicated four-stage LCRA core	141,795	61.0	16.0	160.8	77.2	0.8075
LCRA core, K = 4 (seed 29)	Replicated four-stage LCRA core	141,795	63.2	18.2	206.2	77.2	0.8166
LCRA core, K = 4 (seed 43)	Replicated four-stage LCRA core	141,795	56.8	11.8	177.6	77.2	0.8036
PatchTST (seed 17)	Single-seed deep comparator	122,630	51.0	6.0	32.1	312.9	0.5370
TimesNet (seed 17)	Single-seed deep comparator	1,163,782	59.8	14.8	50.2	175.0	0.7574
iTransformer (seed 17)	Single-seed deep comparator	81,478	64.0	19.0	28.7	169.1	0.7732
TimeMixer (seed 17)	Single-seed deep comparator	83,046	61.8	16.8	56.6	28.4	0.7396
ModernTCN (seed 17)	Single-seed deep comparator	3,612,775	57.5	12.5	72.1	168.3	0.7536
TimeXer (seed 17)	Single-seed deep comparator	101,700	94.5	49.5	63.5	175.7	0.6665
WPMixer (seed 17)	Single-seed deep comparator	168,914	154.0	109.0	97.6	417.8	0.7639
xPatch (seed 17)	Single-seed deep comparator	1,415	160.2	115.2	62.4	147.4	0.7448

Supplementary Table S2. Conservative predictive intervals for the compact one-stage LCRA core ensemble. Coverage, excess above nominal 95% coverage, width, Gaussian NLL, and Gaussian CRPS are reported overall and by member role.
Model and member role	n	Gaussian NLL	Gaussian CRPS	Observed 50% coverage	Observed 80% coverage	Observed 90% coverage	Observed 95% coverage	95% coverage excess	95% width
K = 1 LCRA core ensemble / all members	2973	-1.1599	0.0392	0.716	0.941	0.984	0.994	0.044	0.3977
K = 1 LCRA core ensemble / controls	1982	-1.1604	0.0391	0.722	0.941	0.985	0.994	0.044	0.3980
K = 1 LCRA core ensemble / mapped landforms	991	-1.1589	0.0393	0.705	0.940	0.983	0.995	0.045	0.3970

Supplementary Table S3. Reference-model performance and algebraic consistency for shared pre-event state, phenology, and the S−P and S−I targets.
Target and reference	n	R² or estimate	RMSE or identity error	Purpose
S−P: persistence	2973	0.4036	0.1211	Shared-pre-state and phenology reference
S−P: season-only reference	2973	0.6533	0.0923	Shared-pre-state and phenology reference
S−P: impact EVI + season reference	2973	0.7401	0.0799	Shared-pre-state and phenology reference
S−P: EVI-state + season reference	2973	0.7964	0.0707	Shared-pre-state and phenology reference
S−P: LCRA–iTransformer fusion ensemble	2973	0.8254	0.0655	LCRA–iTransformer fusion ensemble
S−I: persistence	2973	−0.4237	0.1211	Shared-pre-state and phenology reference
S−I: season-only reference	2973	0.2431	0.0883	Shared-pre-state and phenology reference
S−I: impact EVI + season reference	2973	0.3795	0.0799	Shared-pre-state and phenology reference
S−I: EVI-state + season reference	2973	0.5141	0.0707	Shared-pre-state and phenology reference
S−I: LCRA–iTransformer fusion ensemble	2973	0.5897	0.0650	LCRA–iTransformer fusion ensemble
S−I algebraic identity	2973	—	max |error|=2.24e-08	Confirms S−I = (S−P) − (I−P)

Supplementary Table S4. Independent support in the four block-exclusive outer spatial folds.
Outer fold	Blocks	Matched sets	Set-events	Member-events	Unique onsets
1	5	22	263	789	93
2	10	20	238	714	135
3	9	22	276	828	123
4	8	16	214	642	112
Total	32	80	991	2973	281

Supplementary Table S5. Input variables, tensor placement, composite timing, and distinct geometric and physical observation-support definitions. MODIS timing uses 16-day composite starts; Table S7 reports complete-support sensitivity, and Table S8 reports physical 250-m support.
Tensor	Variable	Unit / encoding	Role in prediction
Temporal X: 3×8×8	EVI	scaled product value	Canopy greenness
Temporal X: 3×8×8	valid vegetated-area weight	weighted valid area	Effective valid-area support for polygon-level EVI aggregation
Temporal X: 3×8×8	valid-cell fraction	0–1	Spatial reliability
Temporal X: 3×8×8	sensor	0 Terra / 1 Aqua	Composite identity
Temporal X: 3×8×8	composite-start relative day	days from onset	Product composite-start timing; each composite supports 16 days
Temporal X: 3×8×8	inter-epoch gap	days	Spacing between product composite starts
Temporal X: 3×8×8	pre-window flag	0/1	Window identity
Temporal X: 3×8×8	impact-window flag	0/1	Window identity
Climate-season C: 3×8	simultaneous duration	days	Compound-event duration
Climate-season C: 3×8	maximum heat exceedance	K	Peak thermal stress
Climate-season C: 3×8	accumulated heat exceedance	K days	Integrated thermal stress
Climate-season C: 3×8	soil-moisture deficit	integrated deficit	Water stress
Climate-season C: 3×8	mean affected-area fraction	0–1	Event support
Climate-season C: 3×8	minimum affected-area fraction	0–1	Triplet-wide support
Climate-season C: 3×8	onset DOY sine	cyclic	Seasonal phase
Climate-season C: 3×8	onset DOY cosine	cyclic	Seasonal phase
Static G: 3×8	native geometry-support count	count	Static geometry-support input; distinct from the intersecting MODIS 250-m physical-cell count in Table S8
Static G: 3×8	fractional-area sum	pixel equivalents	Geometry support
Static G: 3×8	mean elevation	m	Terrain
Static G: 3×8	mean slope	degrees	Terrain
Static G: 3×8	mean northness	−1 to 1	Terrain orientation
Static G: 3×8	mean eastness	−1 to 1	Terrain orientation
Static G: 3×8	mapped-landform role	0/1	Matched-member identity
Static G: 3×8	control row index	0/1/2	Explicit member-order encoding with complete-row swap equivariance


Supplementary Table S6. Complete exposed-control balance across all 12 covariates in the final 80-set matched cohort. Signed standardized mean differences compare each mapped landform with the within-triplet mean of its two same-event controls.
Matching covariate	Unit or transform	Sets	Mapped mean (SD)	Control-pair mean (SD)	Signed SMD	|SMD|	|SMD| < 0.10	|SMD| < 0.20
Log polygon area	log-transformed source area	80	13.857 (0.504)	13.857 (0.504)	0.000	0.000	Yes	Yes
Perimeter-to-area ratio	source-derived ratio	80	4.124 (0.815)	4.113 (0.812)	0.013	0.013	Yes	Yes
Primary IGBP-class fraction	fraction (0–1)	80	0.906 (0.090)	0.918 (0.069)	−0.148	0.148	No	Yes
Mean elevation	m	80	1761.866 (870.536)	1738.704 (829.815)	0.027	0.027	Yes	Yes
Mean slope	degrees	80	26.327 (6.542)	27.021 (6.298)	−0.108	0.108	No	Yes
Mean northness	dimensionless (−1 to 1)	80	0.094 (0.540)	0.068 (0.391)	0.054	0.054	Yes	Yes
Mean eastness	dimensionless (−1 to 1)	80	−0.065 (0.625)	−0.035 (0.438)	−0.055	0.055	Yes	Yes
Warm-season 2-m air temperature	K	80	293.605 (4.225)	293.488 (4.102)	0.028	0.028	Yes	Yes
Warm-season soil water, layer 2	m3 m−3	80	0.390 (0.037)	0.390 (0.036)	0.000	0.000	Yes	Yes
Warm-season soil water, layer 3	m3 m−3	80	0.391 (0.040)	0.391 (0.039)	−0.004	0.004	Yes	Yes
Distance to road	log1p(distance in m)	80	5.103 (3.312)	5.154 (3.289)	−0.016	0.016	Yes	Yes
Distance to HydroRIVERS	log1p(distance in m)	80	5.073 (2.777)	5.107 (2.647)	−0.013	0.013	Yes	Yes

Supplementary Table S7. Predictive performance, sampling support, and boundary compliance across cohort and temporal-support specifications. The primary 80-set analysis is compared with complete-support 80-set and 90-set sensitivities under identical spatial folds and seeds.
(A) Cohort, input support, and temporal boundary
Analysis	Scale	Sets	Events	n	Blocks	Onsets	Temporal entries retained/original	Removed	Maximum support-end day	Boundary violations
Primary 80-set nominal-composite analysis	Member level	80	991	2,973	32	281	23,473/23,473	0	46	—
Primary 80-set nominal-composite analysis	Matched contrast	80	991	991	32	281	23,473/23,473	0	46	—
80-set complete-support sensitivity	Member level	80	991	2,973	32	281	17,974/23,473	5,499	31	0
80-set complete-support sensitivity	Matched contrast	80	991	991	32	281	17,974/23,473	5,499	31	0
Complete 90-set complete-support sensitivity	Member level	90	1,117	3,351	33	292	20,266/26,464	6,198	31	0
Complete 90-set complete-support sensitivity	Matched contrast	90	1,117	1,117	33	292	20,266/26,464	6,198	31	0

(B) Predictive accuracy and paired advantages over the linear comparator
Analysis	Scale	Core / linear R²	ΔR² [95% interval]	Core / linear RMSE	RMSE reduction [95% interval]	Core / linear MAE	MAE reduction [95% interval]
Primary 80-set nominal-composite analysis	Member level	0.8219 / 0.7964	0.0254 [0.0037, 0.0532]	0.0662 / 0.0707	0.0046 [0.0007, 0.0086]	0.0513 / 0.0550	0.0037 [0.0003, 0.0076]
Primary 80-set nominal-composite analysis	Matched contrast	0.3684 / 0.3677	0.0007 [−0.0448, 0.0425]	0.0550 / 0.0550	0.0000 [−0.0019, 0.0019]	0.0414 / 0.0415	0.0001 [−0.0015, 0.0019]
80-set complete-support sensitivity	Member level	0.7810 / 0.7768	0.0042 [−0.0304, 0.0351]	0.0734 / 0.0741	0.0007 [−0.0047, 0.0056]	0.0570 / 0.0583	0.0013 [−0.0037, 0.0061]
80-set complete-support sensitivity	Matched contrast	0.2875 / 0.3284	−0.0409 [−0.1226, 0.0240]	0.0584 / 0.0567	−0.0017 [−0.0047, 0.0010]	0.0441 / 0.0428	−0.0012 [−0.0036, 0.0013]
Complete 90-set complete-support sensitivity	Member level	0.7988 / 0.7748	0.0240 [−0.0037, 0.0547]	0.0699 / 0.0739	0.0040 [−0.0006, 0.0088]	0.0541 / 0.0582	0.0041 [0.0002, 0.0087]
Complete 90-set complete-support sensitivity	Matched contrast	0.3342 / 0.3251	0.0091 [−0.0405, 0.0566]	0.0564 / 0.0568	0.0004 [−0.0016, 0.0025]	0.0427 / 0.0427	0.0001 [−0.0017, 0.0021]

Supplementary Table S8. MODIS observation support, response robustness, and within-set event-window structure across matched geometries and events. Physical and Kish-effective support, valid fractions, support–|Δ| relations, support quartiles, and overlapping nominal windows are reported at their corresponding units.
(A) Physical and Kish-effective MODIS support by matched-member role
Role	Support measure	n	Min	Q1	Median	Q3	Max	Mean	SD
Mapped landform	Physical MODIS 250-m cells	80	20	25	28	37	272	36.41	30.64
Mapped landform	Kish-effective MODIS 250-m cells	80	12.53	16.03	18.53	22.42	214.06	24.66	24.63
Matched control	Physical MODIS 250-m cells	160	17	26	28	38	292	36.99	31.11
Matched control	Kish-effective MODIS 250-m cells	160	12.08	16.65	18.36	24.28	234.95	25.26	25.17

(B) Valid-cell fractions across member-events
Role	Valid-cell statistic	Member-events	Min	Q1	Median	Q3	Mean	Count < 0.80
Mapped landform	Minimum across epochs	991	0.826	1.000	1.000	1.000	0.998	0
Mapped landform	Mean across epochs	991	0.986	1.000	1.000	1.000	1.000	—
Matched control	Minimum across epochs	1,982	0.704	1.000	1.000	1.000	0.998	1
Matched control	Mean across epochs	1,982	0.975	1.000	1.000	1.000	1.000	—

(C) Association between observation support and absolute matched response
Endpoint	Weighting	Support measure	n	Blocks	Spearman ρ	95% interval
S−P	Event-equal	Physical MODIS 250-m cells	991	32	0.043	[−0.060, 0.127]
S−P	Event-equal	Kish-effective MODIS 250-m cells	991	32	0.053	[−0.050, 0.134]
S−P	Event-equal	Vegetated mapped area (m²)	991	32	0.049	[−0.057, 0.133]
S−P	Event-equal	Triplet-minimum event-mean valid fraction	991	32	−0.023	[−0.080, 0.028]
S−I	Event-equal	Physical MODIS 250-m cells	991	32	0.042	[−0.107, 0.148]
S−I	Event-equal	Kish-effective MODIS 250-m cells	991	32	0.035	[−0.120, 0.137]
S−I	Event-equal	Vegetated mapped area (m²)	991	32	0.044	[−0.101, 0.141]
S−I	Event-equal	Triplet-minimum event-mean valid fraction	991	32	−0.074	[−0.108, −0.025]
S−P	Set-equal	Physical MODIS 250-m cells	80	32	0.061	[−0.202, 0.251]
S−P	Set-equal	Kish-effective MODIS 250-m cells	80	32	0.103	[−0.174, 0.296]
S−P	Set-equal	Vegetated mapped area (m²)	80	32	0.098	[−0.182, 0.294]
S−P	Set-equal	Triplet-minimum event-mean valid fraction	80	32	−0.447	[−0.678, −0.229]
S−I	Set-equal	Physical MODIS 250-m cells	80	32	0.110	[−0.229, 0.377]
S−I	Set-equal	Kish-effective MODIS 250-m cells	80	32	0.108	[−0.234, 0.362]
S−I	Set-equal	Vegetated mapped area (m²)	80	32	0.119	[−0.209, 0.361]
S−I	Set-equal	Triplet-minimum event-mean valid fraction	80	32	−0.363	[−0.581, −0.147]

(D) Absolute matched response across Kish-effective support quartiles
Endpoint	Weighting	Support quartile	Events	Sets	Blocks	Mean |Δ|	95% interval	Median |Δ|
S−P	Event-equal	Q1 (low)	250	20	16	0.0446	[0.0364, 0.0521]	0.0350
S−P	Set-equal	Q1 (low)	250	20	16	0.0443	[0.0363, 0.0521]	0.0350
S−P	Event-equal	Q2	255	20	12	0.0577	[0.0475, 0.0666]	0.0433
S−P	Set-equal	Q2	255	20	12	0.0569	[0.0477, 0.0652]	0.0433
S−P	Event-equal	Q3	249	20	14	0.0542	[0.0431, 0.0628]	0.0433
S−P	Set-equal	Q3	249	20	14	0.0542	[0.0436, 0.0628]	0.0433
S−P	Event-equal	Q4 (high)	237	20	8	0.0553	[0.0422, 0.0609]	0.0477
S−P	Set-equal	Q4 (high)	237	20	8	0.0555	[0.0416, 0.0615]	0.0477
S−I	Event-equal	Q1 (low)	250	20	16	0.0453	[0.0385, 0.0519]	0.0329
S−I	Set-equal	Q1 (low)	250	20	16	0.0458	[0.0385, 0.0534]	0.0329
S−I	Event-equal	Q2	255	20	12	0.0571	[0.0463, 0.0639]	0.0439
S−I	Set-equal	Q2	255	20	12	0.0572	[0.0469, 0.0643]	0.0439
S−I	Event-equal	Q3	249	20	14	0.0477	[0.0402, 0.0533]	0.0350
S−I	Set-equal	Q3	249	20	14	0.0474	[0.0403, 0.0531]	0.0350
S−I	Event-equal	Q4 (high)	237	20	8	0.0566	[0.0367, 0.0655]	0.0427
S−I	Set-equal	Q4 (high)	237	20	8	0.0565	[0.0351, 0.0659]	0.0427

(E) Chronological overlap among nominal within-set event windows
Measure	Statistic	Estimate	Unit
All within-set event pairs	Count	5822	pairs
Pairs with overlapping 96-day windows	Count	232	pairs
Fraction of pairs with overlap	Fraction	0.0398	fraction
Earlier-target/later-input overlaps	Count	232	pairs
Events involved in any overlap	Count	422	events
Fraction of events involved	Fraction	0.4258	fraction
Sets with any overlap	Count	77	sets
Fraction of sets with overlap	Fraction	0.9625	fraction
Median within-set event fraction involved	Median	0.4286	fraction
Q1 within-set event fraction involved	Q1	0.2863	fraction
Q3 within-set event fraction involved	Q3	0.5385	fraction
Minimum onset separation	Minimum	40	days
Matched-unit fold violations	Count	0	violations
Spatial-block fold violations	Count	0	violations
Overlapping pairs crossing outer folds	Count	0	pairs

Supplementary Table S9. Calendar-standardized vegetation responses and multiplicity-adjusted conditional contrasts across severity and ecological strata. Member-level means share the pooled month-year distribution; event-level sensitivity and all 54 conditional contrasts retain block-by-onset intervals and BH q values.
(A) Member-level severity-quartile marginal means standardized to the pooled month-year distribution
Endpoint	Severity quartile	Member rows	Events	Sets	Blocks	Onsets	Raw mean	Adjusted mean	95% interval
S−P	Q1 (low)	746	297	79	31	120	−0.1433	−0.1107	[−0.1490, −0.0680]
S−P	Q2	742	324	78	32	137	−0.1085	−0.1175	[−0.1522, −0.0731]
S−P	Q3	745	311	79	32	112	−0.0421	−0.0779	[−0.1163, −0.0360]
S−P	Q4 (high)	740	271	80	32	88	−0.0966	−0.0844	[−0.1210, −0.0482]
S−I	Q1 (low)	746	297	79	31	120	−0.0926	−0.0830	[−0.1065, −0.0565]
S−I	Q2	742	324	78	32	137	−0.0483	−0.0565	[−0.0810, −0.0300]
S−I	Q3	745	311	79	32	112	−0.0472	−0.0595	[−0.0802, −0.0381]
S−I	Q4 (high)	740	271	80	32	88	−0.0762	−0.0651	[−0.0893, −0.0401]

(B) Month, year, and seasonal composition within each member-level severity quartile
Severity quartile	Calendar axis	Level	Member rows	Within-quartile proportion	Events	Sets	Blocks	Onsets
Q1 (low)	Month	May	94	0.1260	41	30	9	12
Q1 (low)	Month	June	51	0.0684	22	19	10	14
Q1 (low)	Month	July	158	0.2118	60	48	21	31
Q1 (low)	Month	August	261	0.3499	108	61	23	41
Q1 (low)	Month	September	182	0.2440	66	55	22	22
Q1 (low)	Year	2013	95	0.1273	41	37	13	12
Q1 (low)	Year	2014	41	0.0550	16	16	11	8
Q1 (low)	Year	2015	87	0.1166	36	29	13	13
Q1 (low)	Year	2016	54	0.0724	22	21	14	13
Q1 (low)	Year	2017	66	0.0885	26	23	13	16
Q1 (low)	Year	2018	85	0.1139	35	34	11	11
Q1 (low)	Year	2019	141	0.1890	51	47	14	15
Q1 (low)	Year	2020	6	0.0080	2	2	2	2
Q1 (low)	Year	2022	56	0.0751	22	20	12	12
Q1 (low)	Year	2023	78	0.1046	32	30	10	11
Q1 (low)	Year	2024	37	0.0496	14	14	8	7
Q1 (low)	Season	May–June	145	0.1944	63	39	14	26
Q1 (low)	Season	September	182	0.2440	66	55	22	22
Q1 (low)	Season	July–August	419	0.5617	168	71	27	72
Q2	Month	May	173	0.2332	74	50	18	21
Q2	Month	June	109	0.1469	49	42	18	21
Q2	Month	July	125	0.1685	52	39	23	29
Q2	Month	August	230	0.3100	102	58	20	37
Q2	Month	September	105	0.1415	47	31	21	29
Q2	Year	2013	114	0.1536	51	47	18	15
Q2	Year	2014	45	0.0606	20	19	13	8
Q2	Year	2015	96	0.1294	39	36	12	10
Q2	Year	2016	81	0.1092	39	35	15	17
Q2	Year	2017	43	0.0580	20	18	14	16
Q2	Year	2018	69	0.0930	31	30	16	11
Q2	Year	2019	86	0.1159	33	30	14	13
Q2	Year	2020	31	0.0418	11	11	8	7
Q2	Year	2022	46	0.0620	22	21	13	13
Q2	Year	2023	89	0.1199	39	33	14	16
Q2	Year	2024	36	0.0485	17	15	9	9
Q2	Year	2025	6	0.0081	2	2	2	2
Q2	Season	May–June	282	0.3801	123	63	23	42
Q2	Season	September	105	0.1415	47	31	21	29
Q2	Season	July–August	355	0.4784	154	75	30	66
Q3	Month	May	243	0.3262	104	58	24	23
Q3	Month	June	116	0.1557	50	38	19	22
Q3	Month	July	107	0.1436	40	33	22	22
Q3	Month	August	176	0.2362	73	53	21	24
Q3	Month	September	103	0.1383	44	32	20	21
Q3	Year	2013	78	0.1047	32	29	19	18
Q3	Year	2014	25	0.0336	10	10	6	6
Q3	Year	2015	27	0.0362	16	15	5	6
Q3	Year	2016	138	0.1852	58	48	18	14
Q3	Year	2017	30	0.0403	13	12	10	10
Q3	Year	2018	47	0.0631	19	18	12	7
Q3	Year	2019	16	0.0215	6	6	5	3
Q3	Year	2020	48	0.0644	20	20	10	5
Q3	Year	2022	76	0.1020	31	25	17	15
Q3	Year	2023	73	0.0980	31	28	14	13
Q3	Year	2024	104	0.1396	45	38	17	14
Q3	Year	2025	83	0.1114	30	30	12	1
Q3	Season	May–June	359	0.4819	154	66	27	45
Q3	Season	September	103	0.1383	44	32	20	21
Q3	Season	July–August	283	0.3799	113	68	29	46
Q4 (high)	Month	May	159	0.2149	64	51	25	12
Q4 (high)	Month	June	48	0.0649	19	16	10	10
Q4 (high)	Month	July	156	0.2108	53	49	17	15
Q4 (high)	Month	August	311	0.4203	111	71	31	37
Q4 (high)	Month	September	66	0.0892	24	22	14	14
Q4 (high)	Year	2013	16	0.0216	7	6	6	7
Q4 (high)	Year	2014	12	0.0162	4	4	3	2
Q4 (high)	Year	2015	12	0.0162	6	6	4	1
Q4 (high)	Year	2016	75	0.1014	30	30	16	10
Q4 (high)	Year	2017	32	0.0432	11	11	9	6
Q4 (high)	Year	2018	18	0.0243	7	7	5	4
Q4 (high)	Year	2019	12	0.0162	4	4	4	4
Q4 (high)	Year	2020	35	0.0473	16	16	6	4
Q4 (high)	Year	2022	215	0.2905	73	71	30	24
Q4 (high)	Year	2023	36	0.0486	15	15	7	5
Q4 (high)	Year	2024	192	0.2595	68	63	28	20
Q4 (high)	Year	2025	85	0.1149	30	30	16	1
Q4 (high)	Season	May–June	207	0.2797	83	56	26	22
Q4 (high)	Season	September	66	0.0892	24	22	14	14
Q4 (high)	Season	July–August	467	0.6311	164	80	32	52

(C) Event-equal matched-contrast calendar sensitivity as a distinct estimand
Endpoint	Severity quartile	Events	Sets	Blocks	Onsets	Raw mean	Adjusted mean	95% interval	p analogue
S−P	Q1 (low)	248	78	30	112	0.0020	0.0018	[−0.0106, 0.0145]	0.744
S−P	Q2	248	77	31	113	−0.0025	−0.0047	[−0.0200, 0.0094]	0.542
S−P	Q3	247	79	32	102	0.0051	0.0053	[−0.0072, 0.0177]	0.382
S−P	Q4 (high)	248	80	32	85	0.0027	0.0048	[−0.0092, 0.0190]	0.519
S−I	Q1 (low)	248	78	30	112	0.0058	0.0039	[−0.0104, 0.0165]	0.570
S−I	Q2	248	77	31	113	0.0053	0.0030	[−0.0160, 0.0209]	0.764
S−I	Q3	247	79	32	102	−0.0046	−0.0032	[−0.0171, 0.0120]	0.714
S−I	Q4 (high)	248	80	32	85	0.0029	0.0056	[−0.0086, 0.0188]	0.448
S−P	Q1 (low)	248	78	31	111	−0.0057	−0.0057	[−0.0208, 0.0074]	0.454
S−I	Q1 (low)	248	78	31	111	0.0003	0.0003	[−0.0173, 0.0155]	0.925
S−P	Q2	248	73	29	95	−0.0057	−0.0057	[−0.0209, 0.0083]	0.392
S−I	Q2	248	73	29	95	−0.0027	−0.0027	[−0.0176, 0.0134]	0.810
S−P	Q3	247	79	31	96	0.0123	0.0123	[0.0001, 0.0246]	0.051
S−I	Q3	247	79	31	96	0.0072	0.0072	[−0.0082, 0.0213]	0.344
S−P	Q4 (high)	248	73	32	102	0.0064	0.0064	[−0.0064, 0.0179]	0.289
S−I	Q4 (high)	248	73	32	102	0.0045	0.0045	[−0.0061, 0.0160]	0.435

(D) Complete 54-comparison family of event-equal conditional contrasts
Stratum family	Stratum	Endpoint	Support E/S/B/O	Mean contrast	95% interval	p analogue	BH q
Season	May–June	S−P	331/80/32/77	0.0072	[−0.0093, 0.0203]	0.350	0.943
Season	May–June	S−I	331/80/32/77	0.0007	[−0.0135, 0.0151]	0.897	0.986
Season	July–August	S−P	508/80/32/150	−0.0006	[−0.0104, 0.0122]	0.971	0.992
Season	July–August	S−I	508/80/32/150	0.0032	[−0.0077, 0.0133]	0.527	0.943
Season	September	S−P	152/70/30/54	−0.0019	[−0.0218, 0.0155]	0.837	0.973
Season	September	S−I	152/70/30/54	0.0031	[−0.0125, 0.0178]	0.716	0.943
Pre-event canopy	Q1 (low)	S−P	248/71/29/99	0.0197	[−0.0002, 0.0401]	0.054	0.439
Pre-event canopy	Q1 (low)	S−I	248/71/29/99	0.0004	[−0.0149, 0.0141]	0.913	0.986
Pre-event canopy	Q2	S−P	248/70/29/139	0.0091	[−0.0041, 0.0251]	0.152	0.746
Pre-event canopy	Q2	S−I	248/70/29/139	0.0033	[−0.0101, 0.0183]	0.626	0.943
Pre-event canopy	Q3	S−P	247/68/29/143	−0.0018	[−0.0166, 0.0118]	0.832	0.973
Pre-event canopy	Q3	S−I	247/68/29/143	−0.0003	[−0.0148, 0.0129]	0.992	0.992
Pre-event canopy	Q4 (high)	S−P	248/56/24/120	−0.0198	[−0.0365, −0.0032]	0.021	0.378
Pre-event canopy	Q4 (high)	S−I	248/56/24/120	0.0058	[−0.0114, 0.0211]	0.510	0.943
Severity	Q1 (low)	S−P	248/78/30/112	0.0020	[−0.0094, 0.0147]	0.681	0.943
Severity	Q1 (low)	S−I	248/78/30/112	0.0058	[−0.0078, 0.0181]	0.356	0.943
Severity	Q2	S−P	248/77/31/113	−0.0025	[−0.0187, 0.0122]	0.716	0.943
Severity	Q2	S−I	248/77/31/113	0.0053	[−0.0150, 0.0228]	0.617	0.943
Severity	Q3	S−P	247/79/32/102	0.0051	[−0.0084, 0.0188]	0.440	0.943
Severity	Q3	S−I	247/79/32/102	−0.0046	[−0.0189, 0.0102]	0.584	0.943
Severity	Q4 (high)	S−P	248/80/32/85	0.0027	[−0.0090, 0.0147]	0.624	0.943
Severity	Q4 (high)	S−I	248/80/32/85	0.0029	[−0.0090, 0.0157]	0.663	0.943
Spatial block	B003_003	S−P	181/15/1/53	0.0009	[−0.0119, 0.0121]	0.847	0.973
Spatial block	B003_003	S−I	181/15/1/53	0.0043	[−0.0127, 0.0181]	0.557	0.943
Spatial block	B003_002	S−P	144/11/1/48	0.0018	[−0.0097, 0.0125]	0.738	0.944
Spatial block	B003_002	S−I	144/11/1/48	0.0015	[−0.0121, 0.0134]	0.836	0.973
Spatial block	B003_004	S−P	66/5/1/47	−0.0005	[−0.0154, 0.0140]	0.962	0.992
Spatial block	B003_004	S−I	66/5/1/47	0.0140	[−0.0006, 0.0278]	0.057	0.439
Spatial block	B008_004	S−P	43/3/1/29	0.0119	[−0.0064, 0.0279]	0.182	0.819
Spatial block	B008_004	S−I	43/3/1/29	0.0058	[−0.0134, 0.0246]	0.515	0.943
Spatial block	B002_003	S−P	42/3/1/30	−0.0105	[−0.0389, 0.0169]	0.465	0.943
Spatial block	B002_003	S−I	42/3/1/30	−0.0205	[−0.0486, 0.0063]	0.147	0.746
Spatial block	B004_002	S−P	42/3/1/20	−0.0043	[−0.0310, 0.0238]	0.686	0.943
Spatial block	B004_002	S−I	42/3/1/20	−0.0171	[−0.0382, 0.0011]	0.075	0.506
Spatial block	B009_003	S−P	37/3/1/21	−0.0040	[−0.0147, 0.0080]	0.496	0.943
Spatial block	B009_003	S−I	37/3/1/21	−0.0068	[−0.0247, 0.0078]	0.435	0.943
Spatial block	B004_009	S−P	35/3/1/17	0.0124	[0.0005, 0.0215]	0.040	0.432
Spatial block	B004_009	S−I	35/3/1/17	0.0092	[0.0041, 0.0142]	<0.001	0.054
Spatial block	B003_005	S−P	33/3/1/26	0.0043	[−0.0163, 0.0275]	0.624	0.943
Spatial block	B003_005	S−I	33/3/1/26	0.0104	[−0.0104, 0.0284]	0.247	0.889
Spatial block	B008_003	S−P	27/2/1/23	−0.0157	[−0.0462, 0.0074]	0.199	0.826
Spatial block	B008_003	S−I	27/2/1/23	−0.0061	[−0.0343, 0.0245]	0.673	0.943
Spatial block	B013_005	S−P	26/2/1/21	−0.0086	[−0.0206, 0.0018]	0.117	0.702
Spatial block	B013_005	S−I	26/2/1/21	−0.0017	[−0.0136, 0.0101]	0.752	0.944
Spatial block	B012_004	S−P	25/2/1/15	0.0211	[0.0057, 0.0427]	0.003	0.081
Spatial block	B012_004	S−I	25/2/1/15	0.0188	[0.0014, 0.0396]	0.031	0.418
Spatial block	B003_009	S−P	24/2/1/18	−0.0028	[−0.0425, 0.0343]	0.909	0.986
Spatial block	B003_009	S−I	24/2/1/18	0.0124	[−0.0217, 0.0474]	0.459	0.943
Spatial block	B009_002	S−P	24/2/1/19	0.0003	[−0.0192, 0.0220]	0.983	0.992
Spatial block	B009_002	S−I	24/2/1/19	0.0098	[−0.0129, 0.0356]	0.428	0.943
Spatial block	B014_001	S−P	22/2/1/13	0.0048	[−0.0136, 0.0231]	0.598	0.943
Spatial block	B014_001	S−I	22/2/1/13	0.0146	[−0.0069, 0.0371]	0.219	0.844
Spatial block	B012_006	S−P	20/2/1/15	0.0078	[−0.0091, 0.0230]	0.331	0.943
Spatial block	B012_006	S−I	20/2/1/15	−0.0058	[−0.0223, 0.0098]	0.442	0.943
